# Direct Detection of Unamplified Pathogen RNA in Blood Lysate using an Integrated Lab-in-a-Stick Device and Ultrabright SERS Nanorattles

**DOI:** 10.1038/s41598-018-21615-3

**Published:** 2018-03-06

**Authors:** Hoan T. Ngo, Elizabeth Freedman, Ren Abelard Odion, Pietro Strobbia, Agampodi Swarnapali De Silva Indrasekara, Priya Vohra, Steve M. Taylor, Tuan Vo-Dinh

**Affiliations:** 1 Fitzpatrick Institute for Photonics, Duke University, Durham, NC 27708 USA; 2 Department of Biomedical Engineering, Duke University, Durham, NC 27708 USA; 3 Department of Medicine & Duke Global Health Institute, Duke University, Durham, NC 27708 USA; 4 Division of Head and Neck Surgery and Communication Sciences, Duke University, Durham, NC 27708 USA; 5 Department of Chemistry, Duke University, Durham, NC 27708 USA; 6 Present Address: Biomedical Engineering Department, International University, Vietnam National University-Ho Chi Minh City (VNU-HCMC), Ho Chi Minh City, Vietnam

## Abstract

Direct detection of genetic biomarkers in body fluid lysate without target amplification will revolutionize nucleic acid-based diagnostics. However, the low concentration of target sequences makes this goal challenging. We report a method for direct detection of pathogen RNA in blood lysate using a bioassay using surface-enhanced Raman spectroscopy (SERS)-based detection integrated in a “lab-in-a-stick” portable device. Two levels of signal enhancement were employed to achieve the sensitivity required for direct detection. Each target sequence was tagged with an ultrabright SERS-encoded nanorattle with ultrahigh SERS signals, and these tagged target sequences were concentrated into a focused spot for detection using hybridization sandwiches with magnetic microbeads. Furthermore, the washing process was automated by integration into a “lab-in-a-stick” portable device. We could directly detect synthetic target with a limit of detection of 200 fM. More importantly, we detected *plasmodium falciparum* malaria parasite RNA directly in infected red blood cells lysate. To our knowledge, this is the first report of SERS-based direct detection of pathogen nucleic acid in blood lysate without nucleic acid extraction or target amplification. The results show the potential of our integrated bioassay for field use and point-of-care diagnostics.

## Introduction

Nucleic acid-based molecular diagnostics are of paramount importance in medical diagnostics owing to their high specificity and sensitivity as well as their ability to genotype targets, among other advantages. Many nucleic acid detection assays for molecular diagnostics have been proposed that employ target amplification or/and signal amplification. However, to date, polymerase chain reaction (PCR) remains the gold standard for nucleic acid tests, which are mostly conducted in diagnostic laboratories owing to the complexity of PCR procedure^1^. Recent outbreaks of Zika virus and Ebola showed that the ability to detect nucleic acid targets of pathogens at the point-of-care (POC) or in austere settings is highly desired. Rapid, accurate, POC nucleic acid detection devices can help rapidly identify infected patients at the site of care, thus preventing diseases from spreading and informing interventional procedures. Such devices are highly desired not only in developing countries but also in the developed world.

Signal amplification assays offer specific operational advantages compared to traditional PCR-based target amplification assays as described in recent review papers^2–4^. While target amplification assays are sensitive, they require target extraction and purification, complex reaction mixtures, and are prone to false-positives. In contrast, signal amplification assays typically do not require nucleic acid purification and target amplification steps, thus simplifying the detection workflow. Furthermore, signal amplification assays can avoid both target loss resulting from nucleic acid purification and imprecise quantification resulting from biases in target amplification. Finally, signal amplification assays do not require enzymatic amplification and are therefore more resistant to inhibitors and less prone to false positives. However, signal amplification assays also have limitation. Compared to PCR-based target amplification assays, most existing signal amplification assays have lower sensitivity. Many efforts have been devoted to develop ultrasensitive signal amplification assays for detection of unamplified nucleic acid targets^2–11^. In most of these works (except for^5^ and^11^), however, nucleic acid was purified prior detection, and direct detection of nucleic acid targets in blood lysate was not demonstrated.

SERS is an appealing candidate for signal amplification assays. SERS is a phenomenon in which Raman scattering of molecules adsorbed on metallic nanostructures is enhanced millions of times or more^12,13^, and in prior studies has proved to be a sensitive analytical technique with single molecule sensitivity^14,15^. Based on SERS, different chemical and biological sensing assays for *in vitro* and *in vivo* medical diagnostics have been reported^16–18^. SERS-based nucleic acid detection has attracted a lot of interest^19–49^ our group reported a sensitive nanorattle-based sandwich assay, in which the presence of synthetic nucleic acid targets in hybridization buffer was detected by sandwich hybridization of magnetic beads functionalized with capture probes and ultrabright SERS-encoded nanorattles functionalized with reporter probes^50^. However, to the best of our knowledge, SERS-based direct detection of nucleic acid targets in un-manipulated blood lysate has not been reported. Such capability will simplify the sample preparation process, which is the true bottle neck in nucleic acid-based molecular diagnostics at the POC or low-resource settings^51^. However, to the best of our knowledge, SERS-based direct detection of nucleic acid targets in un-manipulated blood lysate has not been reported. Such capability will simplify the sample preparation process, which is the true bottle neck in nucleic acid-based molecular diagnostics at the POC or low-resource settings.

In this manuscript, we developed a new class of ultrabright SERS-encoded nanorattles based on nanocubes, referred to as cube nanorattles, and employed them for direct detection of unamplified malaria RNA in blood lysate. The cube nanorattles have core-gap-shell structure in which Raman reporters are loaded in the gap space between the core and the shell, thus improving SERS signal stability. We then utilized the cube nanorattles for nucleic acid detection based on sandwich hybridization. In comparison to the SERS-based single-step nucleic acid assays^38–40,43^, sandwich hybridization requires washing to remove the unreacted components and non-specific bindings. Manual washing is laborious and not suitable for POC and automatic washing using microfluidics requires pumps and valves for fluid manipulation, which adds complexity and cost. To meet the need for simple but automated washing, we herein present the concept of a “lab-in-a-stick”, in which sequential washes are integrated in a portable device for automatic washing. The portable device includes an aluminum heat block with closed-loop temperature control and a handheld Raman reader for SERS measurement. Experimental results showed 200 fM limit of detection with synthetic targets, which was equivalent to 2 attomoles given the tested sample volumes. Furthermore, we demonstrated that the combination of the cube nanorattle-based sandwich assay and the lab-in-a-stick portable device successfully detected real RNA targets extracted from the malaria parasite *Plasmodium falciparum*. Finally, as a proof-of-concept, SERS-based direct detection of *P*. *falciparum* RNA in malaria-infected RBC lysate was demonstrated for the first time. Further development and optimization of the nanorattle-based sandwich assay and the lab-in-a-stick portable device have potential to produce a “sample-in-answer-out” molecular diagnostic device that can be used in the field and at the POC.

## Results and Discussion

### SERS-encoded cube nanorattle synthesis and characterization

**Figure 1:**
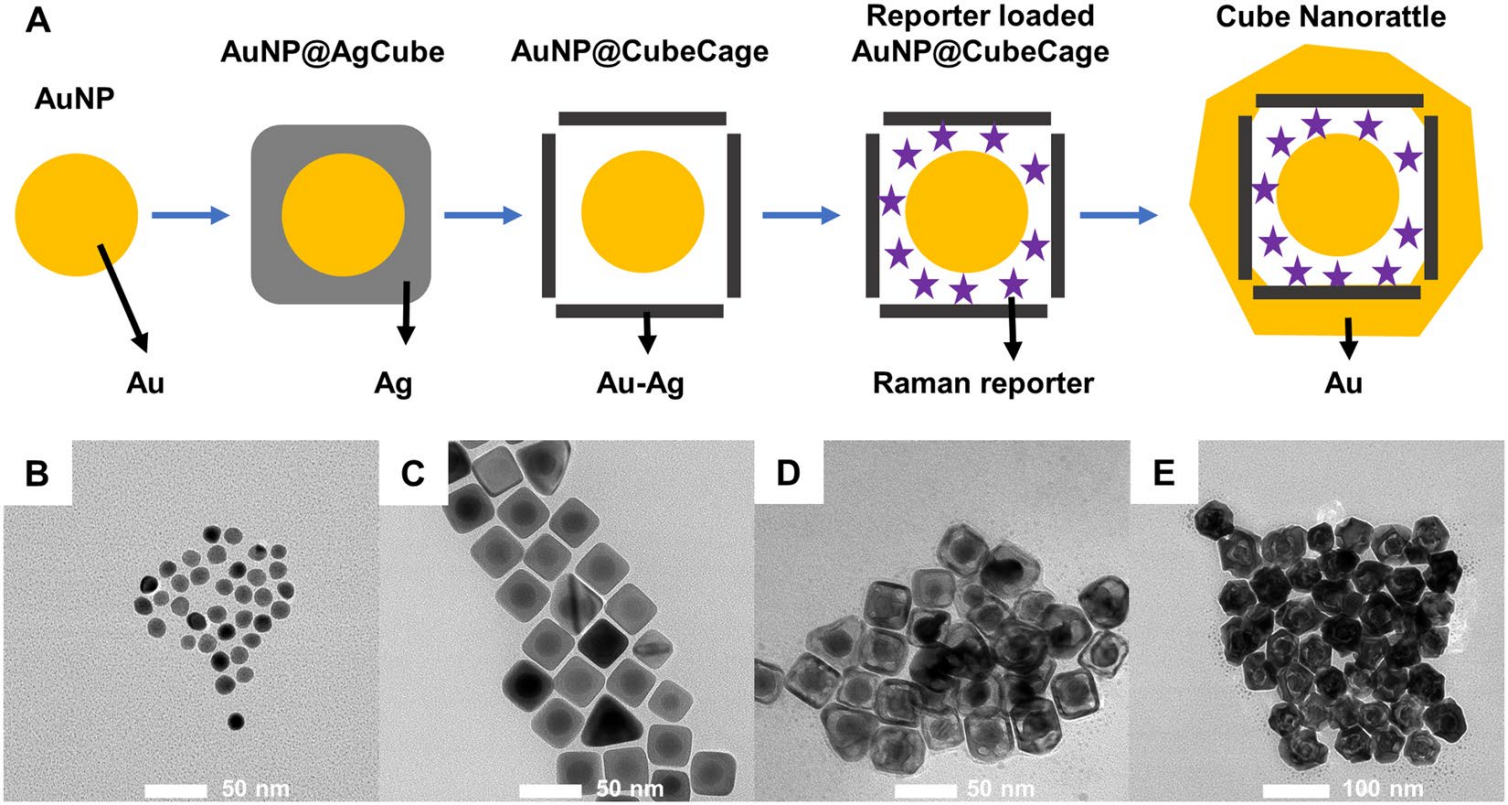
(**A**) Cubic nanorattle synthesis process. TEM images of (**B**) AuNP; (**C**) AuNP@AgCube; (**D**) Reporter loaded AuNP@CubeCage; (**E**) Cube nanorattles.

Figure 1A shows different steps of the cube nanorattles synthesis process, which was developed based on previous works^52,53^. First, single-crystal, spherical gold nanoparticle (AuNP) cores were synthesized by a seed-mediated method^52^. The AuNP cores were then coated with cubic Ag shells to obtain AuNP@AgCube. Galvanic replacement^54^ was used to transform cubic Ag shells into cubic Au-Ag cages containing AuNP in the interior (AuNP@CubeCage). Raman reporters were loaded into the space between AuNP cores and porous cubic cages. After loading Raman reporters, the porous cubic cages were turned into complete shells by a final Au coating to obtain cube nanorattles.

TEM and SEM images of the intermediate and final products of SERS-encoded cube nanorattles are shown in Fig. 1B–E. Figure 1B shows 14 nm AuNP which were subsequently grown into 20 nm AuNP (not shown). The 20 nm AuNP were coated with cubic Ag shells to obtain AuNP@AgCube (Fig. 1C). The edge length of cubic Ag shells was 34 ± 2 nm. It is noteworthy that the successful formation of cubic shape of the Ag shells strongly depended on the crystallography of AuNP cores. When single-crystal AuNP cores synthesized using growth solution containing CTAC and high ascorbic acid concentration as described in^52^ were used for Ag coating, mostly cubic, single-crystal Ag shells was obtained (Fig. S1A). This could be explained by the higher growth rate of Ag on the {111} facets than on the {100} facets of this kind of AuNP cores, resulting AuNP@AgCube dominated by the {100} facets^52^. When AuNP cores synthesized using growth solution containing CTAC and low ascorbic acid concentration as described in^55^ were used, mostly spherical, single-crystal Ag shells were obtained (Fig. S1B). Similarly, when polycrystalline AuNP cores synthesized using citrate method were used, mostly spherical, polycrystalline Ag shells were obtained (Fig. S1C and D).

Using galvanic replacement, AuNP@AgCube were turned into AuNP@CubeCage composed of cubic Au-Ag cages with AuNP in the interior (Fig. 1D). The cubic Au-Ag cages had edge length of 37 ± 3 nm and wall thickness of 3.5 ± 0.5 nm. The cubic Au-Ag cages were porous with holes at corners of the cubes (Fig. S5), indicating that the Ag dissolution started at corners of cubic Ag shells of the AuNP@AgCube. This could be explained by the poor passivation, provided by poly(vinyl pyrrolidone) (PVP) - a stabilizing polymer present in the reaction mixture, of the {111} facets at the corners in comparison to passivation of the {100} facets on side surfaces of cubic Ag shells^56^. The existence of these holes allowed Raman reporters to diffuse into the gap space between AuNP cores and porous cubic cages to obtain reporter-loaded AuNP@CubeCage (not shown). After Raman reporter loading, the porous cubical cages were turned into complete shells by a final Au coating to obtain SERS-encoded cube nanorattles (Fig. 1E). The size of nanorattles was 54 ± 6 nm. The role of the final Au coating step was to prevent Raman reporters from leaking out of nanorattles, thus improving SERS signal stability. In a previous work, our group has demonstrated that nanorattles with final Au coating have stable SERS signal in cellular system for 24 h^57^.

**Figure 2:**
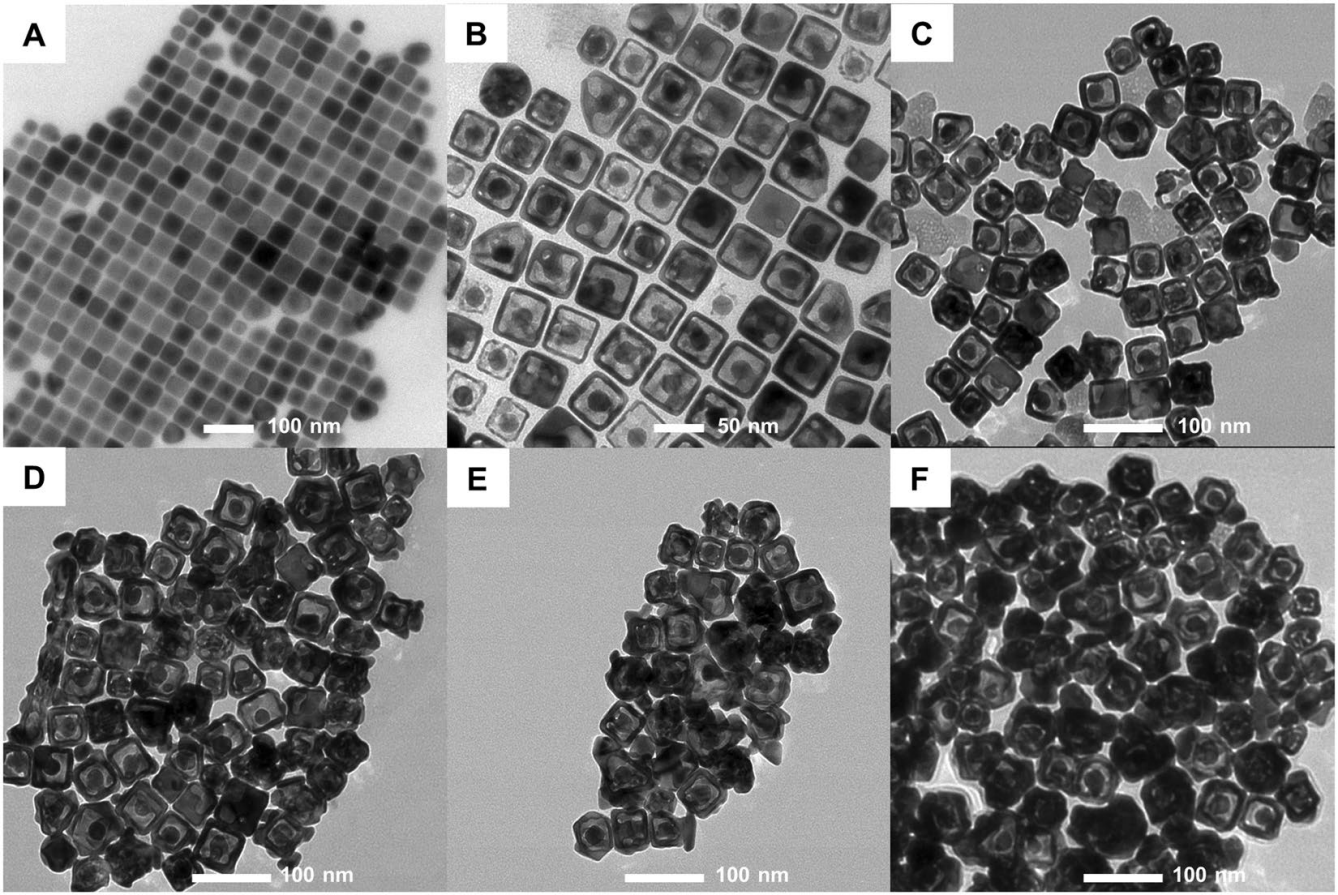
TEM images of (**A**) AuNP@AgCube, (**B**) AuNP@CubeCage, (**C**) Cube Nanorattle-50, (**D**) Cube Nanorattle-100, (**E**) Cube Nanorattle-200, (**F**) Cube Nanorattle-400. The numbers indicate volume (µL) of 5 mM Au^3+^ used for final gold coating in 10 ml batches.

To investigate the relationship between the amount of Au coating to nanorattles’ SERS signal stability, nanorattles with different amounts of final Au coating were suspended in 10% hydrogen peroxide, and SERS intensity was monitored along the time. Nanorattles coated with higher amount of Au showed a slower rate of reduction of SERS signal (Fig. S2), thus having better SERS signal stability. We therefore expected our SERS-encoded cube nanorattles, with final Au coating, to have better SERS signal stability than SERS-encoded nanorattles without final Au coating previously reported in^53^. It is noteworthy that higher Au coating amount resulted in thicker shells (Fig. 2), preventing more light from reaching and exciting Raman reporters residing in the interior of nanorattles, thus resulting in lower SERS intensity (Fig. S3). There is therefore a trade-off between SERS signal stability and intensity. In this work, the thickness of the final Au coating was adjusted to ~10 nm to achieve SERS signal stability in cellular system^57^ while still exhibiting bright SERS intensity.

**Figure 3:**
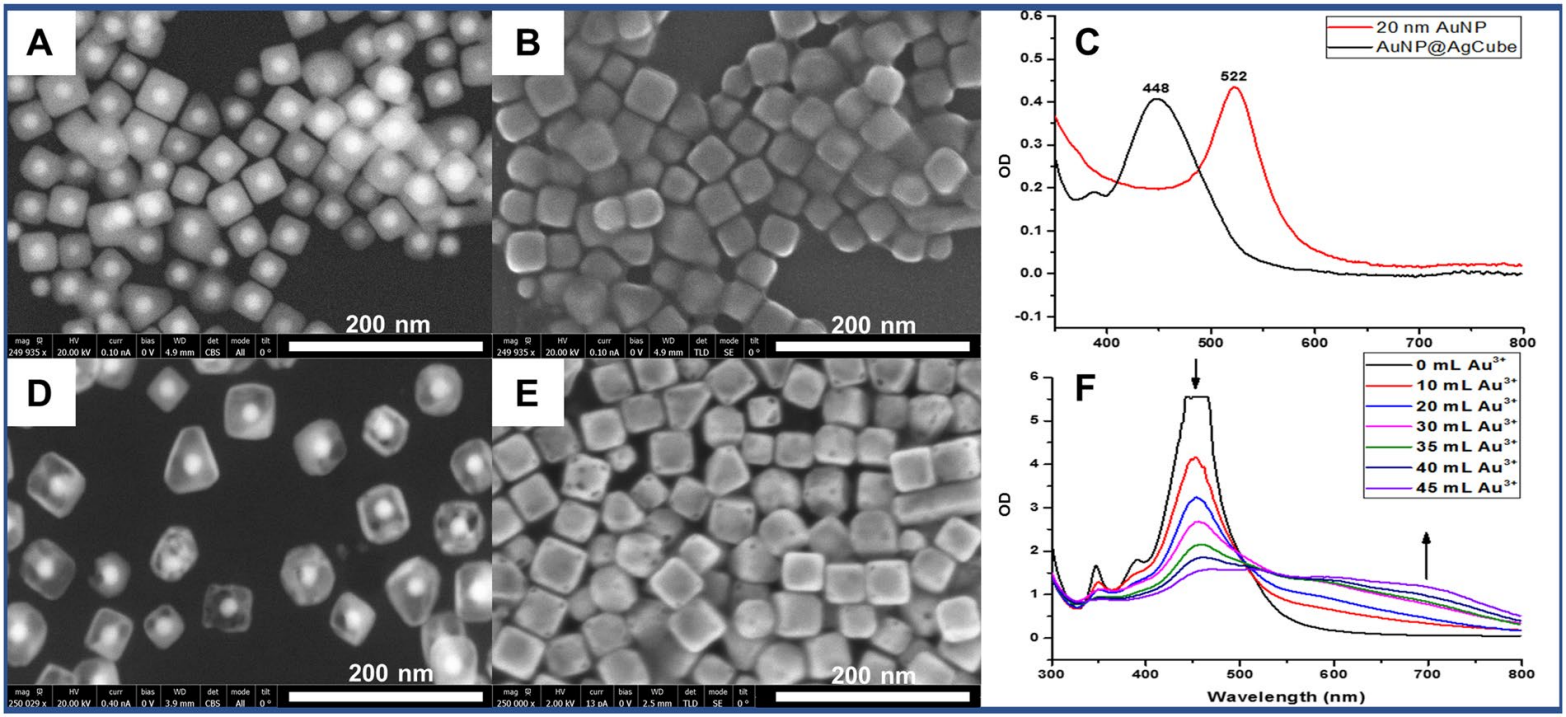
SEM images (**A**,**B**) and optical extinction spectra (**C**) of AuNP@AgCube; SEM images (**D**,**E**) and optical extinction (**F**) of AuNP@CubeCage; (**A**) and (**D**) were acquired in back scattering mode.

SEM images and optical extinction spectra of AuNP@AgCube and AuNP@CubeCage are shown in Fig. 3. Using the backscattered electron imaging mode of SEM (FEI Verios 460 L), spherical AuNP cores inside of AuNP@AgCube and AuNP@CubeCage could be clearly seen (Fig. 3A,D, respectively). In addition, while Ag shells of the AuNP@AgCube are solid (Fig. 3A), Au-Ag shells of the AuNP@CubeCage are hollow (Fig. 3D). Backscattered electron imaging mode of SEM provides alternative to TEM to look at inner structures of nanoparticles. Conveniently, upon switching to the common secondary electron imaging mode of SEM, the exterior of AuNP@AgCube and AuNP@CubeCage can be seen (Fig. 3B,E, respectively). While Fig. 3B confirms the cubic shape of AuNP@AgCube, Figs 3E and S5 show holes at the corners of AuNP@CubeCage. As previously mentioned, the existence of the holes allowed Raman reporters to be loaded into the interior of AuNP@Cage.

Optical extinction spectra of AuNP and AuNP@AgCube are shown in Fig. 3C. While AuNP has a localized surface plasmon resonance (LSPR) peak at 522 nm, AuNP@AgCube has LSPR peak blue shifted to 448 nm. The disappearance of AuNP peak in AuNP@AgCube extinction indicates that Ag shells are thick enough to screen AuNP resonance. Figure 3F shows the evolution of LSPR of AuNP@AgCube during galvanic replacement reaction. When total amount of Au^3+^ added to the reaction is increased, the 448 nm peak decreases, indicating that Ag shells were continuously being dissolved. Simultaneously, the extinction in near-infrared region increased. The increase is attributed to the formation of hollow cages as the galvanic replacement reaction proceeds. At the end of the galvanic replacement reaction, LSPR peak at 522 nm reappeared, indicating that the hollow shell of AuNP@CubeCage was no longer thick enough to screen LSPR of AuNP residing inside the AuNP@CubeCage. The shoulder at around 448 nm was likely due to residual Ag in bimetallic Au-Ag hollow shell of the AuNP@CubeCage.

**Figure 4:**
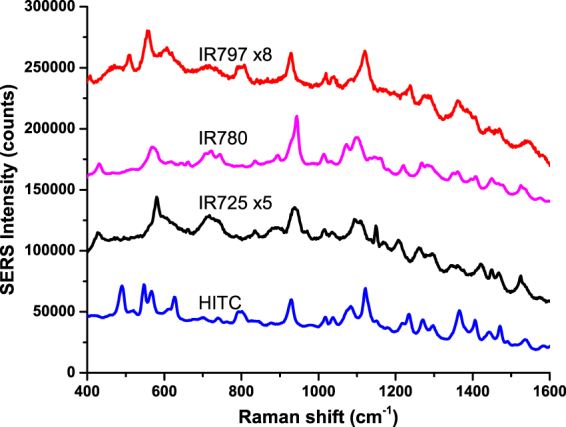
SERS spectrum of SERS-encoded cube nanorattles loaded with different Raman reporters.

Upon loading Raman reporters into AuNP@CubeCage followed by final Au coating, cubic nanorattles were obtained. Figure 4 shows nanorattles loaded with different Raman reporters, thus possessing distinctive spectral fingerprints. Among four Raman reporters, HITC and IR780 yielded higher SERS intensities than IR725 and IR797. Employing principal component analysis^58^, these nanorattles can be easily differentiated (Fig. S5). Compared to gold nanostars (AuNS), another SERS-encoded nanoparticles developed in our group, cubic nanorattles showed ~5 times higher SERS intensity with HITC reporter and ~3 higher with IR780 reporter (Fig. S7). In contrast to AuNS where reporters simply adsorb on AuNS surface – thus being more prone to reporter desorption and signal degradation, nanorattles well protect the Raman reporters inside their complete metal shells, resulting in highly stable SERS intensity as discussed above.

It is noteworthy that, in this work, two different methods to load Raman reporters into AuNP@CubeCage, with and without the employment of phase-change material 1-tetradecanol, were investigated. In the method employing tetradecanol^59^, Raman reporters were trapped inside AuNP@CubeCage. Raman reporter-loaded AuNP@CubeCage can thus be washed several times without significant Raman reporter loss during washing. Multiple washing helped to remove the excess reporters on AuNP@CubeCage outer surface, resulting in relatively uniform final Au coating (Fig. 2). In another method, AuNP@CubeCage were simply mixed with Raman reporters in solution at room temperature for several hours without tetradecanol. Raman reporters attached to inner and outer surfaces of AuNP@CubeCage due adsorption. The AuNP@CubeCage were then washed once followed by final Au coating. Although this Raman reporter loading method was simpler, the uniformity of final Au coating varied depending on Raman reporters (Fig. S7). While HITC and DTTC Raman reporters resulted in uneven Au coating (Fig. S7A and B), IR780 and IR792 resulted in nice uniform Au coating (Fig. S7C and D).

### Lab-in-a-stick concept

**Figure 5:**
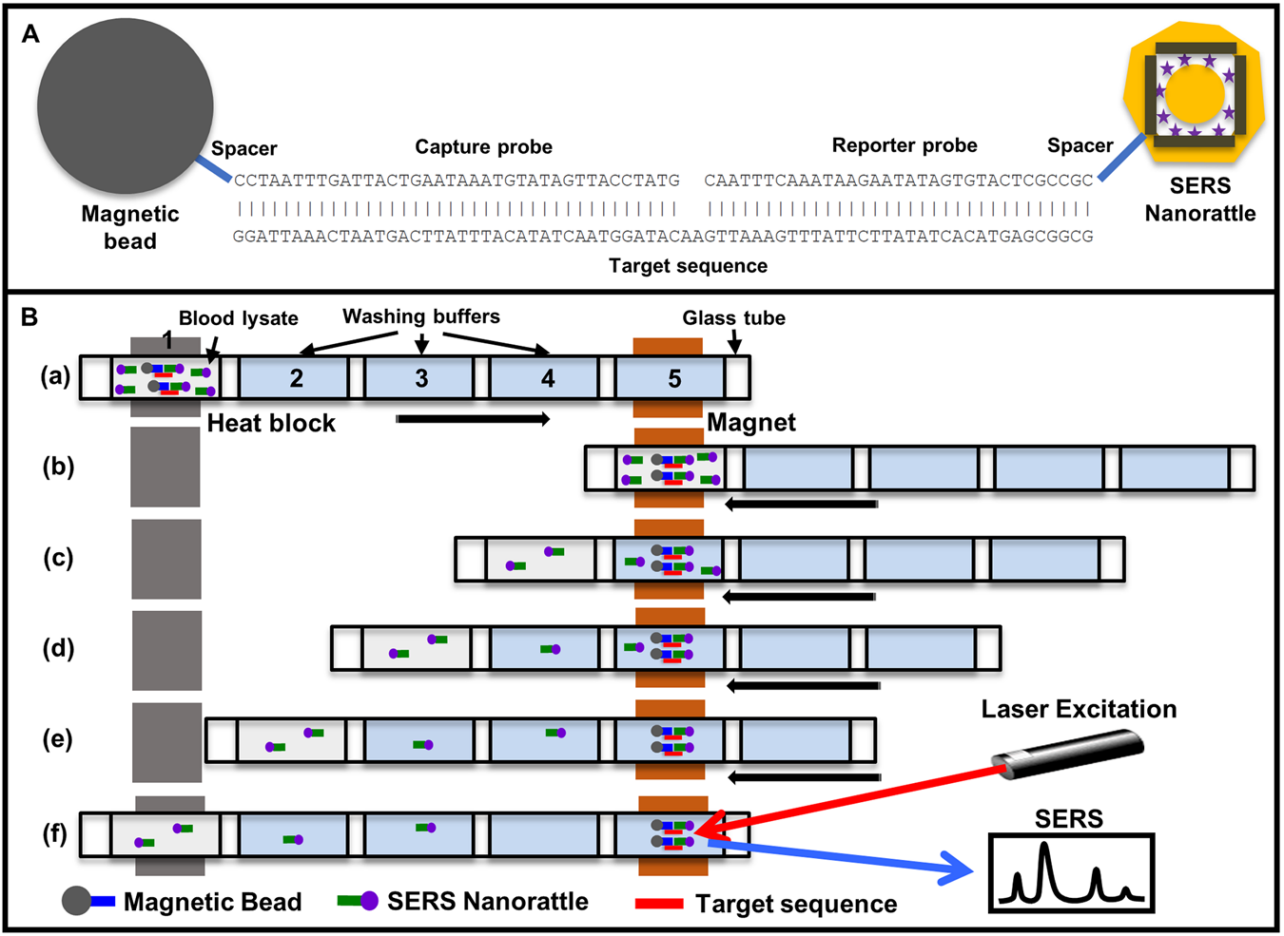
(**A**) The nanorattle-based DNA detection method using sandwich hybridization of magnetic bead that are loaded with capture probes, target sequence, and ultrabright SERS-encoded nanorattles that are loaded with reporter probes. (**B**) Lab-in-a-stick concept.

The detection scheme of the nanorattle-based sandwich assay is shown in Fig. 5. Magnetic beads and nanorattles are functionalized with DNA capture probes and DNA reporter probes, respectively. The two probes are designed to be complementary to the two ends of the target sequences. In the presence of the target sequences, hybridization sandwiches of magnetic bead-target sequence-nanorattle are formed (Fig. 5A). To wash away the unreacted components in automatic fashion, “lab-in-a-stick” concept was developed (Fig. 5B) based on previous works^60,61^. All reagents are loaded into glass capillary tubes/sticks. Solution 1 is a mixture of magnetic beads functionalized with DNA capture probes, target sequences, and SERS-encoded cube nanorattles functionalized with DNA reporter probes in hybridization buffer. Solution 2, 3, and 4 are washing buffers. Solution 5 is buffer solution for SERS measurement. PCR-grade mineral oil was used to separate the solutions. In step (a), target sequences are captured by magnetic beads and labeled by SERS-encoded nanorattles in hybridization mixture 1. Upon hybridization sandwich formation, the glass tube is moved to the right so that the hybridization mixture is right above permanent magnet. The hybridization sandwiches are pulled down by the magnet and form a pellet as shown in step (b). The tube is then slowly moved back to the original position during which the pellet of hybridization sandwiches is dragged through the washing solutions 2, 3, and 4, leaving unreacted nanorattles and non-specific binding behind as shown in step (c), (d), and (e). Finally, in step (f), SERS signal is measured by focusing laser onto the pellet of hybridization sandwiches in measurement buffer 5.

The use of a permanent magnet for pulling magnet beads through different solutions loaded inside a tube have been previously reported by the Haselton group^60–62^ and several other groups^63–67^. Besides permanent magnets, electromagnets have also been used^68^. The movement of magnetic beads in these works were however mainly for nucleic acid purification. Negatively-charged nucleic acids in sample lysate are bound onto positively-charged silica magnetic beads through charge interaction. The nucleic acid-bound silica magnetic beads are then moved through different washing buffer to remove non-specifically bound impurities. The purified nucleic acid was detected using enzymatic amplification such as PCR or loop-mediated isothermal amplification (LAMP). Wang *et al*. used magnetic beads conjugated with antibody to capture CD4 cells in unprocessed whole blood. The CD4-bound magnetic beads were then moved through different solutions for washing and enzyme-linked immunosorbent assay (ELISA) detection^67^. Gottheil *et al*. reported a similar concept for interleukin-8 detection using fluorescence readout^66^. To the best of our knowledge, the herein presented employment of pulling magnetic beads through a tube for washing nucleic acid hybridization sandwiches has not been reported.

Washing off the excess nanorattles and non-specific bindings from hybridization sandwiches is critical to achieve low blank signals (background), thus lowering limit of detection. The lab-in-a-stick concept enables automatic washing of hybridization sandwiches without the need of microfluidics which often require fluid manipulation using pumps and valves. The washing process was accomplished by simply pulling preloaded-loaded tube back and forth relative to a permanent magnet. This is a step forward in comparison to the laborious and time-consuming manual washing that was previously used in which hybridization complexes were concentrated by a permanent magnet or a magnet stand followed by manual pipetting (Fig. S10). Lab-in-a-stick washing is thus more suitable for POC applications. In the following section, we will describe how we integrated the concept into a portable device that was designed for employment at the field.

### Lab-in-a-stick integrated device

**Figure 6:**
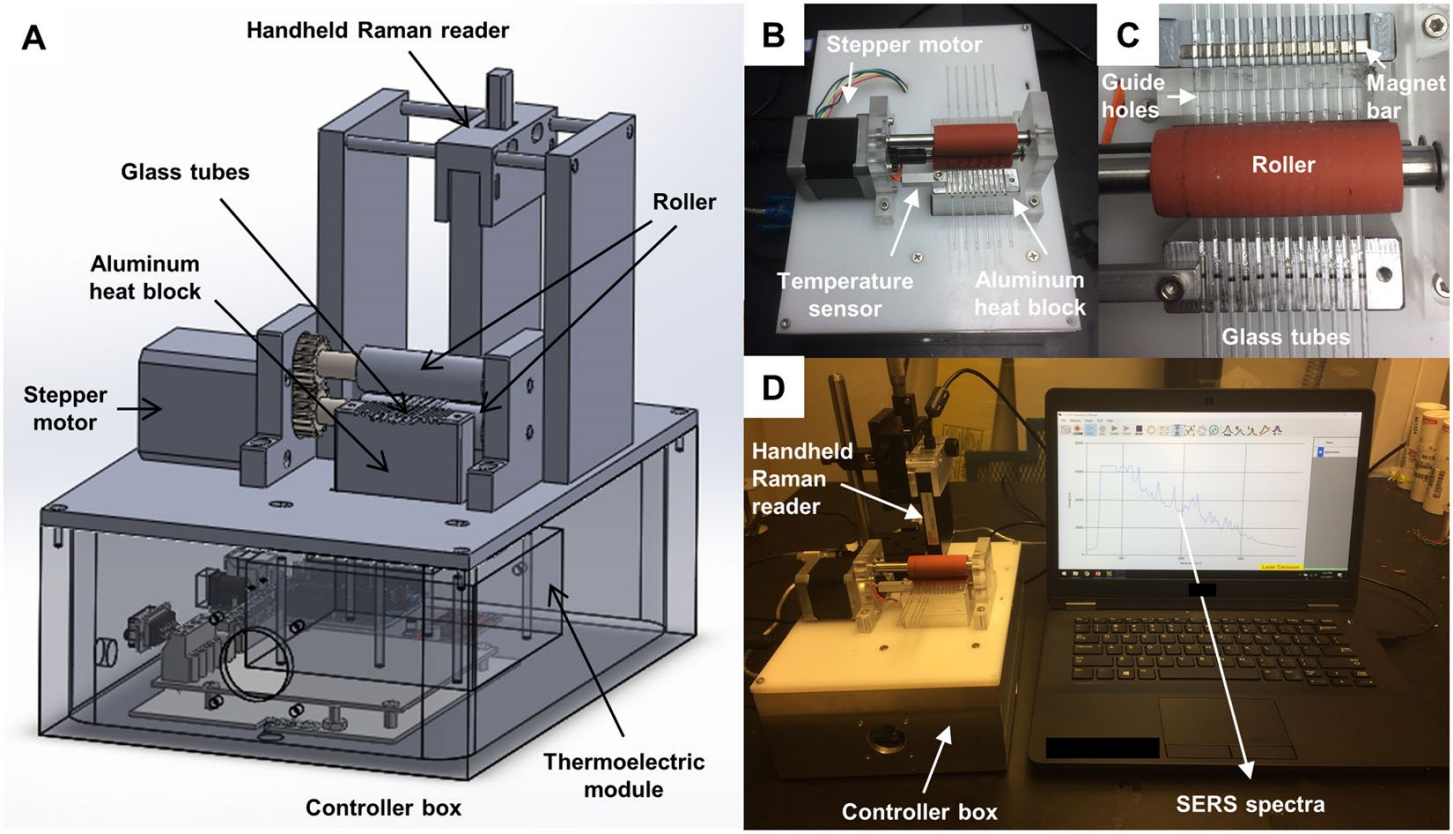
(**A**) 3D model of the lab-in-a-stick device. (**B**–**D**) The lab-in-a-stick portable device. The device that can handle up to twelve samples simultaneously with temperature control.

The design and images of the portable device integrating the lab-in-a-stick concept are shown in Fig. 6. Since nucleic acid-based diagnostics is usually implemented with each sample having multiple replicates in addition to negative and/or positive controls, we designed the device to be able to process multiple samples simultaneously (up to twelve). SolidWorks 3D model of the device is shown in Fig. 6A. A stepper motor was used to drive two horizontal rollers, which in turn drive the glass tubes that were sandwiched between the two rollers. To ensure that the tubes move straight, their movement were guided by the guide holes and the grooves on aluminum heat block (Fig. 6B,C). Compared to the tube pulling design by the Haselton group^61^, our design can be readily scaled up to afford more tubes by simply increasing rollers’ length, aluminum heat block’s size, and the number of guide holes. The aluminum heat block was connected to a thermoelectric module positioned under the heat block (Fig. 6A) and a temperature sensor (Fig. 6B), allowing closed-loop temperature control. A permanent magnet bar was placed after the guide holes and under the tubes (Fig. 6C) for concentrating and moving hybridization sandwiches inside the tubes. The use of a permanent magnetic bar has several advantages including strong magnetic field, no energy consumption, and no heat dissipation. SERS signal was measured by focusing laser beam from the handheld Raman reader Hamamatsu Photonics K.K., Japan, Fig. 6D) onto the hybridization sandwiches concentrated by the permanent magnet. It is noteworthy that when target sequence concentration was in pM range or lower, the handheld Raman reader was not sensitive enough, and a lab-built Raman fiber probe-based system^50^ that is mounted a wheeled cart was used instead. All electronics components were housed inside the controller box (Fig. 6A,D). A LabVIEW program was developed to control the stepper motor which drives the glass tube. Another LabVIEW program (TE Technology, MI, USA) was used to control temperature.

Operational procedure of the device is quite simple. First, glass tubes were placed onto the device as shown in Fig. 6C. Washing solutions and sample solutions (e.g. blood lysate) spiked with functionalized magnetic beads and nanorattles were loaded into the glass tubes by pipetting. All solutions were separated to each other by 2 µL mineral oil spacers. The solutions were positioned so that the sample solutions were on top of the heat block (Fig. 5B, step a). The heat block temperature was increased to 40 °C. Sample solutions were incubated at this temperature for several hours for hybridization sandwich formation. During this time, the tubes were continuously moved back and forth in short distances to decrease the magnetic bead sedimentation rate. After that, the glass tubes were pulled toward the permanent magnet by the two rollers for magnetic bead concentration (Fig. 5B, step b). The glass tubes were then slowly pulled back to the original position by the two rollers. Hybridization complexes were thus dragged through washing solutions, leaving excess nanorattles and non-specific binding behind (Fig. 5B, step c to e). In each of the washing solutions, the magnetic beads were agitated by quickly moving the tube back and forth in short distances multiple times to remove non-specific binding. Finally, laser beam was focused on the hybridization sandwiches for SERS measurement (Fig. 5B, step f).

### Detection of synthetic DNA target and extracted RNA in hybridization buffer

**Figure 7:**
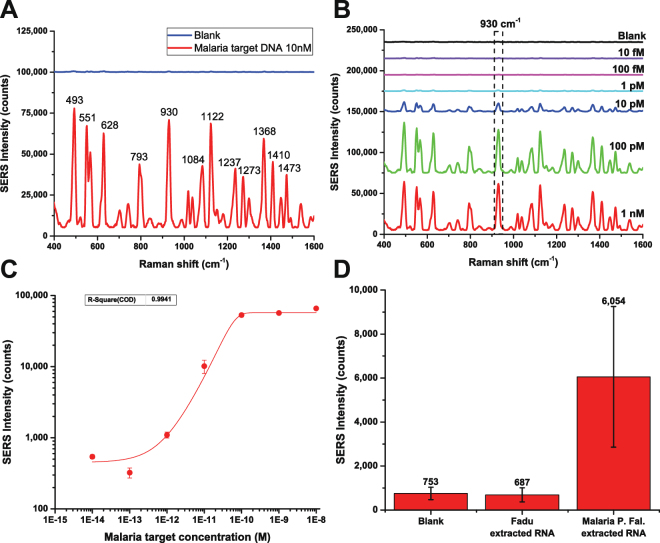
Detection of malaria synthetic DNA and malaria extracted RNA. (**A**) SERS spectra of 10 nM malaria synthetic DNA vs. Blank. (**B**) SERS spectra of blank and 10 fM to 1 nM malaria synthetic DNA. (**C**) SERS intensity at the 930 cm^−1^ peak at different malaria synthetic DNA concentration. (**D**) Direct detection of malaria P. falciparum RNA extracted from red blood cells infected with malaria parasites. Error bars represent standard deviation from (n = 3). In addition, the Supplementary Information section provides the spectra for concentrations below 10 pM from (**B**) plotted in scale (Fig. S11) and a table with the values and error of the data points shown in (**C**) (Table S1).

Using the lab-in-a-stick integrated device, we conducted a quantitative test using serial dilutions of malaria synthetic target sequences in hybridization buffer. The target sequence was chosen from a highly conserved region of the 18S rRNA gene of *P*. *falciparum*; this RNA sequence is highly expressed during the asexual (blood) stage of P. falciparum, accounting for up to 20% of all RNA sequences present in blood-stage parasites^69^. Capture probe and reporter probe were designed to be complementary to the two ends of the target sequence (Fig. 5A). SERS-encoded cube nanorattles loaded with 1,1′,3,3,3′,3′-hexamethylindotricarbocyanine iodide (HITC) were used as the SERS nanotags. SERS spectra of samples with different synthetic target concentrations ranging from 10 nM to 10 fM and a blank are shown in Fig. 7A,B. SERS peaks of the HITC-loaded cube nanorattles are labeled in Fig. A, and the SERS intensities at 930 cm^−1^ in Fig. B were used to plot the calibration curve (Fig. 7C). Using the IUPAC standard method (LOD = y_blank_ + 3 × SD_blank_, y_blank_ and SD_blank_ is the average and standard deviation of the blank signals), limit of detection was calculated to be 200 femtomolar. This was equivalent to 2 attomoles, given the tested volume of each sample 10 μL. The linear dynamic range of the assay ranges approximately from 1 pM to 100 pM.

Experiments with real biological samples, RNA extracted from cultured malaria parasites, were also conducted. SERS intensities from the blank (negative control), RNA extracted from a human cell line (Fadu, negative control), and RNA extracted from cultured malaria parasite (positive control) are shown in Fig. 7D. While the signal from the blank and the human cell RNA were low, the signal from the malaria RNA was high, indicating that our nanorattle-based sandwich method could specifically and directly detect RNA extracted from malaria parasite without target amplification.

### Direct detection of unamplified malaria RNA in blood lysate

**Figure 8:**
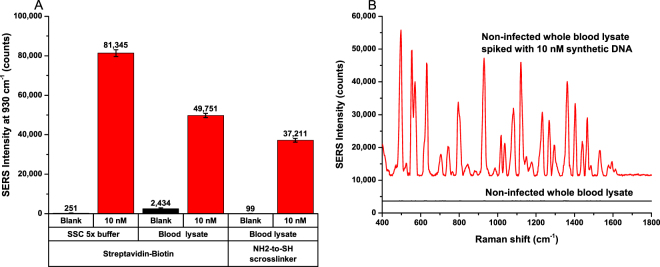
(**A**) Performance comparison between two different magnetic bead-capture probe conjugation chemistries: streptavidin-biotin and amine-to-thiol crosslinker. (**B**) Detection of 10 nM synthetic malaria DNA spiked into whole blood lysate. Error bars represent standard deviation from (n = 3).

In this section, we tested performance of the assay in blood lysate. We started with an experiment to detect synthetic targets that were spiked into blood lysate. QuantiGene sample processing kit for blood samples (Thermofisher) was used to lyse healthy blood samples, followed by the addition of synthetic targets (10 nM final concentration). Magnetic beads functionalized with capture probes and nanorattles functionalized with reporter probes were added to the blood lysates (spiked with synthetic targets). The mixtures were loaded into lab-in-a-stick device for incubation, automatic washing, and detection as described earlier. The results showed that synthetic target at 10 nM could still be detected in blood lysate as shown in Fig. 8A. However, in this experiment, when streptavidin magnetic beads functionalized with biotinylated capture probes using streptavidin-biotin interaction were used, we observed a significant increase in blank signal when the assay was tested in blood lysate in comparison to in hybridization buffer. This can be attributed to potential denaturation of streptavidin on magnetic bead’s surface by various components in blood lysate, resulting in more non-specific bindings and high blank signal. In contrast, for amine magnetic beads functionalized with thiolated capture probes using a NH_2_-to-SH crosslinker (sulfosuccinimidyl 4-(N-maleimidophenyl)butyrate), we achieved a particularly low blank background signal in blood lysate (below 100 counts, Fig. 8A). Meanwhile, the signal for 10 nM targets spiked into blood lysate was still quite intense (more than 37,000 counts). SERS spectra of these samples are shown in Fig. 8B. The results indicated that the amine magnetic bead and NH_2_-to-SH crosslinker are more suitable for blood lysate experiments in comparison to streptavidin magnetic bead and streptavidin-biotin interaction.

**Figure 9:**
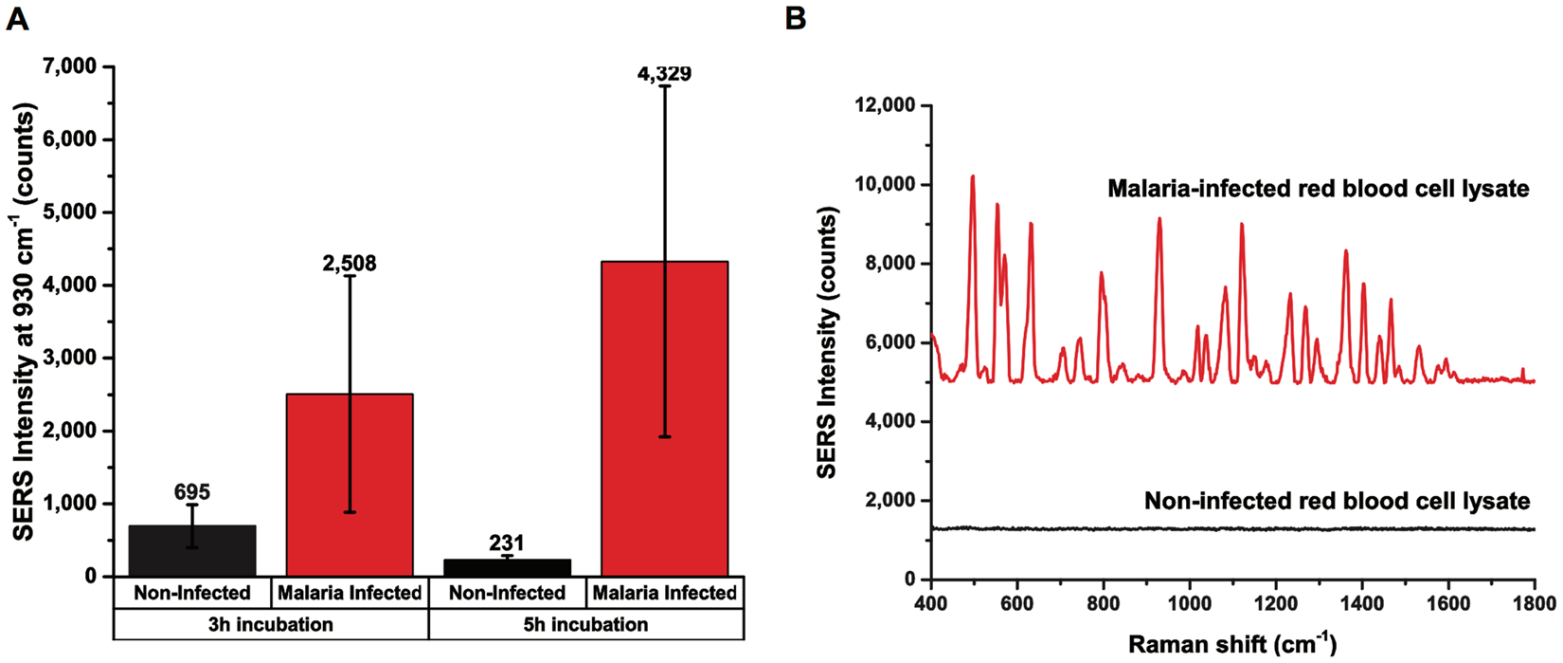
(**A**) Direct detection of malaria RNA in lysate of malaria-infected red blood cells using 3 h and 5 h incubation time. (**B**) SERS spectrum of direct detection of malaria RNA in lysate of malaria-infected red blood cells using 5 h incubation. Error bars represent standard deviation from (n = 3).

Finally, direct detection of native malaria RNA in lysate of cultured malaria-infected red blood cells (RBSc) was conducted. RBCs infected with P. falciparum strain FVO was cultured by the method described by Trager and Jensen^70^. The cultured malaria-infected RBCs samples was determined to be at 4% parasite density as quantified by smear microscopy. Assuming 10 million erythrocytes per microliter of packed erythrocytes, 4% parasite density would yield 400,000 infected RBCs per microliter. Malaria-infected RBCs lysate was prepared using the QuantiGene sample processing kit for blood samples. Amine magnetic beads functionalized with capture probes and ultrabright SERS-encoded cube nanorattles functionalized with reporter probes were used for the detection of 18s rRNA targets in malaria-infected RBCs lysate. Without any nucleic acid extraction or target amplification such as PCR, we observed a clear difference between the SERS intensity of malaria-infected RBCs samples and of non-infected RBCs samples (Fig. 9A). Longer incubation time (5 hours) resulted in higher signals than 3 hours incubation. Long incubation time could be explained by low concentration of malaria RNA targets, resulting in slow sandwich formation kinetic. In the future, the incubation time could potentially be reduced by employing active mixing of magnetic beads in blood lysate.

Average SERS spectra of malaria-infected RBCs samples vs. healthy non-infected RBCs samples using 5 h incubation are shown in Fig. 9B. Direct detection of pathogen nucleic acid in blood lysate without purification and target amplification is particularly challenging due to low target concentration (fM range or lower) and harsh blood lysate environment. Herein, we demonstrated for the first time that the cube nanorattle-based sandwich assay integrated in a lab-in-a-stick portable device could be used for direction detection of malaria RNA in lysate of cultured malaria-infected RBCs without any RNA extraction or target amplification. It is noteworthy that the malaria-infected RBCs used in this study had relatively high malaria parasite density. In the future, the assay and the device can be further optimized for detection of malaria-infected RBCs with lower, more clinical relevant malaria parasite densities.

## Conclusion

We have synthesized novel ultrabright SERS-encoded cube nanorattles. Raman reporters were protected inside the nanorattles, making the them highly stable in biological systems. Using the ultrabright SERS cube nanorattles and magnetic beads, we have developed a sensitive DNA detection assay based on and sandwich hybridization. A lab-in-a-stick portable device was developed for the automation of the washing process. Using the device, up to twelve samples could be simultaneously washed in automatic fashion. The results with synthetic target sequences showed a limit of detection of 200 femtomolar (2 attomole). Furthermore, this device was capable of directly detecting malaria RNA extracted from malaria parasites without target amplification. Finally, we demonstrated proof-of-concept SERS-based direct detection of malaria RNA in malaria-infected RBCs lysate without any RNA extraction or target amplification. The detection process was simple and involved adding nanorattles pre-functionalized with reporter probes and magnetic beads pre-functionalized with capture probes to blood lysate samples followed by several hour incubation, an automated washing step using a lab-in-a-stick portable device, and SERS measurement. To the best of our knowledge, this is the first time a SERS-based direct detection of pathogen nucleic acid in blood lysate without any nucleic acid extraction or target amplification was reported.

## Materials and Methods

### Materials

Sodium borohydride (NaBH_4_), hexadecyltrimethylammonium bromide (CTAB), Au chloride solution (HAuCl_4_) 200 mg/dL in deionized water (ref # HT1004-100ML Sigma), ascorbic acid (AA), hexadecyltrimethylammonium chloride (CTAC) solution 25 wt. % in water (ref # 292737 Aldrich), polyvinylpyrrolidone Mw ~55,000 (PVP), methanol (MetOH), 1,3,3,1′,3′,3′,-hexamethyl-2,2′-indotricarbocyanine iodide (HITC), tetradecanol (TD), phosphate buffer saline (PBS), tris-EDTA buffer solution (TE), tween 20, sodium chloride (NaCl), hydrochloric acid (HCl) were purchased from Sigma-Aldrich. Sodium citrate dihydrate was purchased from BDH. Methoxy polyethylene glycol thiol Mw 5000 (mPEG-SH) was purchased from Nanocs. Magnetic beads were purchased from Life Technologies. All DNA sequences (Table S1) were synthesized by Integrated DNA Technologies (IDT, Coralville, IA). Millipore Synergy ultrapure water (DI) of resistivity = 18.2 MΩ cm was used in all nanoparticle synthesis solutions. Nuclease-free water was used in all experiments relating to RNA and DNA.

### AuNP synthesis

AuNP were synthesized using a seed- mediated method^52^. Au seeds were prepared by adding 0.6 ml of 10 mM ice cold NaBH_4_ to 10 ml of 0.25 mM Au chloride (HAuCl_4_) in 0.1 M CTAB solution under vigorous stirring. Upon NaBH_4_ addition, the solution’s color quickly changed from yellow to brown, indicating the formation of small Au seeds. Stirring was stopped after 10 min and the seed solution was aged for 3 h at 27 °C to ensure complete decomposition of remaining NaBH_4_. Growth solution was prepared by adding 3 mL of 1.0 M freshly prepared AA to mixture of 3.94 mL of Au chloride (~5.08 mM concentration), 10.58 mL of CTAC solution, and 92.4 mL of DI water. Upon AA addition, the Au chloride solution quickly changed color from yellow to colorless, indicating the reduction of Au3^+^ to Au^+^. AuNP (~14 nm) was synthesized by adding 2 mL Au seeds to the above growth solution under magnetic stirring. Solution color changed from colorless to wine red within a few minutes, indicating the growth of Au seeds into larger AuNP. After the solution color became stable, magnetic stirring was stopped, and the solution was left undisturbed for 1 hour. Growth solution for synthesizing 20 nm AuNP was prepared by adding 7.5 mL of 1.0 M freshly prepared AA to mixture of 10 mL of Au chloride (~5.08 mM concentration), 52.9 mL of CTAC solution, and 479.5 mL of DI water. AuNP (~20 nm) was synthesized by adding 20 mL of 14 nm AuNP to the above growth solution under magnetic stirring. The solution was stirred for five minutes and left undisturbed overnight in dark.

AuNP’s extinction coefficient was measured using FLUOstar Omega microplate reader. Extinction coefficient measurements showed LSPR peak at ~522 nm. For transmission electron microscope (TEM) imaging, 5 µL of the prepared AuNP was dropped on Formvar carbon film-coated 200 mesh copper grids and allowed to dry at room temperature. TEM images were taken using FEI Tecnai G² Twin TEM system. TEM image analysis using ImageJ showed that AuNP have diameter of ~20 nm.

### Ag shell coating and galvanic replacement

Two hundred twenty-five mL of the as-prepared AuNP (20 nm) were washed once with DI (12,500 rcf, 20 minutes) and resuspended in equal volume of 20 mM CTAC. The solution was heated to 60 °C for 20 minutes under magnetic stirring. Ag cubic shells were coated on 20 nm AuNP to form Au-Ag core-shell structures (AuNP@AgCube). Briefly, 100 mL of 2 mM AgNO_3_ and 100 mL of 50 mM AA in 40 mM CTAC were simultaneously added to the heated AuNP solution at the rate of 5 mL/min/each solution. The process took 20 minutes during which the color slowly changed from light red to orange. The mixture was covered with parafilm M (Bemis) and left at 60 °C for 4 hour under magnetic stirring to obtain AuNP@AgCube.

AuNP@AgCube (270 mL) were centrifuged once at 5,000 rcf for 15 m followed by resuspending in mixture of 135 mL of 100 mM PVP and 135 mL of 0.2 M CTAC. The solution was heated to 90 °C for 2 min before 35 mL–45 mL of 0.5 mM Au chloride solution was slowly added. The process took approximately 20 minutes. During Au chloride addition, the solution color changed from orange to dark brown, and finally to purple, indicating that the Ag shells were galvanically replaced by Au3^+^ to turn into porous Au-Ag cages. The solution, containing porous Au-Ag cages with AuNP inside (AuNP@CubeCage), was further heated for 10 min and then cooled down to room temperature. AuNP@CubeCage were collected by centrifugation (6,000 rcf, 20 minutes) and resuspensded in DI water to remove excess PVP and CTAC. The resuspension was centrifuged once more time and resuspended in 6 mL DI water to obtain stock AuNP@CubeCage solution.

### Loading Raman reporters into AuNP@CageCube

For Raman reporter loading without using TD, fifty µL of stock AuNP@CageCube were mixed with 100 µL of 1 mM Raman reporters in 850 µL of EtOH. The mixture was left at room temperature for 2 hours under shaking followed by centrifugation at 7,000 rpm for 10 minutes. The pellet was washed once with DI and resuspended in 1 mL of 0.1 M CTAC.

For Raman reporter loading using TD, the AuNP@Cage in MetOH solution was heated to 50 °C in a glass bottle followed by addition of 200 mg tetradecanol and 20 µL of HITC dye. The solution was heated overnight to completely evaporate MetOH and to allow tetradecanol and HITC dyes to go into AuNP@Cage structures. Boiling DI water (3 mL) was added to separate phases, one being TD/dye mixture and the other containing water and a small amount of AuNP@Cage. The majority of AuNP@Cages were however stuck on the glass bottle bottom. The solution was kept in an ice bath for 2 mins to solidify TD. After TD solidification, sonication was used to re-disperse AuNP@Cages stuck at the glass bottle bottom into water, turning the solution color to purple. The mixture was set aside for 5 mins to allow TD to form a layer on the solution’s surface. The solution, without TD, was carefully transferred into a 15 mL centrifuge tube using a pipette. DI water was added to increase the solution volume to 10 mL. The solution was centrifuged and resuspended in 1 mM CTAC solution several times to remove excess TD and dyes.

### Final Au coating

Growth solution was prepared by adding 500 µL of 0.2 M AA (freshly prepared) to mixture of 150 µL Au chloride solution (~5.08 mM) and 8.5 mL of 0.1 M CTAC. Raman reporter-loaded AuNP@CageCube prepared above (1 mL in 0.1 M CTAC) were added to the growth solution under magnetic stirring. Stirring was stopped after a few minutes, and the solution was left undisturbed overnight in dark. Nanocube nanorattles were obtained by centrifuging the solution at 3,000 rcf for 15 minutes followed by washing the pellet once with DI water and resuspension in 1 mL DI water.

### Functionalization of nanorattles with thiolated DNA Reporter Probes

Nanorattles were functionalized with DNA reporter probes using a pH-assisted method with slight modifications^50^. First, 5 µL of 100 mM TCEP in TE 1X was added to 50 μL of 100 μM thiolated DNA reporter probes. The mixture was incubated for 1 h at room temperature before being added to 1 mL of nanorattles. The new mixture was incubated for 1 hour under shaking. Then 10 μL of citrate-HCl buffer (300 mM trisodium citrate, pH adjusted to 3.1 using 1 M HCl) was added to promote loading of DNA onto nanorattles. One hour later, the mixture was centrifuged at 6,500 rpm for 5 minutes. Fifty microlitters of 1 mM SH-PEG 5 K (freshly prepared, sonicated 5 minutes) was added to the pellet followed by addition of 1 mL PBS 1X with 0.01% TW20 and sonication. The solution was centrifuged at 6,500 rpm for 5 minutes. The pellet was washed once with TE 1X followed by resuspension in TE 1X and storage at 4 °C before use. The functionalized nanorattles are good for use within 3–4 months.

### Functionalization of streptavidin magnetic bead with biotinylated DNA capture probes

Magnetic beads (Dynabeads MyOne Streptavidin C1, 1 μm diameter) were functionalized with DNA capture probes using the manufacturer’s protocol. Briefly, 200 μL of 10 mg/mL stock magnetic bead was washed three times using 1 mL washing buffer 1X (5 mM Tris-HCl pH 7.5, 0.5 mM EDTA, 1 M NaCl) and resuspended in 400 μL washing buffer 2X. To load DNA capture probes on magnetic beads, 400 μL of 5 μM biotinylated DNA capture probe was added to the resuspension. The mixture was placed at room temperature under gentle shaking for 0.5 h. Then, DNA capture probe-loaded magnetic beads were washed three times using washing buffer 1X and resuspended in 1600 μL TE 1X (magnetic bead final conc. 1.25 mg/ml). The as-prepared capture probe-loaded magnetic bead solution was stored at 4 °C and used within 3–4 months.

### Functionalization of amine magnetic bead with thiolated DNA capture probes using amine-to-thiol crosslinker

A published protocol^71^ with some modifications was used to functionalize amine magnetic bead with thiolated DNA capture probes. Dynabeads M-270 Amine (MMPs), amine-to-thiol crosslinker sulfo-SMPB (sulfosuccinimidyl 4-(N-maleimidophenyl)butyrate), sulfo-NHS-acetate, and TCEP·HCl (Tris(2-carboxyethyl)phosphine hydrochloride) were obtained from Thermofisher. Dimethyl sulfoxide anhydrous (DMSO) was obtained from Sigma-Aldrich. Passivation buffer contained 150 mM phosphate buffer and 150 mM NaCl (pH = 7.5). Coupling buffer contained 100 mM phosphate buffer and 200 mM NaCl (pH = 6.9–7.0).

MMPs was removed from refrigerator ~15 min before use. Two hundred microliters of MMPs was washed three times with 1.5 mL DMSO (be sure to remove all supernatant). Ten milligrams of SMPB was dissolved in 200 μL DMSO. Add the SMPB-DMSO solution to the dry MMPs pellet and transfer the MMP-SMPB solution into a 10 mL tube. The SMPB bottle and MMP tube were washed with DMSO, and all DMSO washes were added to the 10 mL tube. Volume of solution in the 10 mL tube was made up to 3.16 mL using DMSO. The mixture was wrapped with aluminum foil and placed on shaker for 4 hours. After 4 hours, the MMPs were washed three times with 15 mL DMSO (tube was changed between second and third wash, cover particles with foil while separating). Wash the MMPs twice with coupling buffer, resuspend in 1 mL coupling buffer, transfer to 1.5 mL microcentrifuge tube, remove supernatant. TCEP-reduced thiolated DNA capture probes were prepared by adding 5 μL of 0.1 M TCEP to 50 μL of 100 µM thiolated DNA capture probes. The solution was incubated for 45 min before being added to the MMPs, wrap in foil, and shake for 1 hour at room temperature. Remove supernatant, resuspend in 1 mL coupling buffer, transfer to a new 1.5 mL tube, wash three times with 1 mL coupling buffer (change the tube between second and third wash).

For passivation, wash the MMPs twice with 1 mL passivation buffer, change to 10 mL tube between first and second washes. Dissolve 20 mg sulfo-NHS-acetate in 7 mL passivation buffer. Add this solution to the dry MMPs. Wrap in foil and shake for 1 hour at room temperature. Wash the MMPs three times with 10 mL passivation buffer, changing tubes between second and third washes. Wash twice with 1 mL storage buffer (TE 1X), with final resuspension in 1 mL storage buffer (TE 1X). Store at 4 °C.

### Nucleic acid detection in hybridization buffers

DNA quantification capability of the nanorattle-based method was demonstrated by testing sample solutions containing synthetic DNA sequences of wild type malaria parasite P. falciparum at different concentrations. Sample solutions were prepared by serial dilution of stock target sequence in hybridization buffer (SSC 5X, BSA 1%, SDS 0.02%, freshly prepared). RNA extracted from malaria parasite P. falciparum was also tested by adding 1 μL extracted RNA to 4 μL hybridization buffer. For each 5 μL sample solution, 2 μL of magnetic beads loaded with capture probes and 3 μL of nanorattles loaded with reporter probes were added. The mixtures were pipetted into glass capillary tubes placed on the second-generation lab-on-a-stick device. After 3 h of incubation at 40 °C, hybridization complexes were washed three times (100 cycles each) in three washing buffers (same composition as the hybridization buffer). The hybridization complexes were then moved to the fourth washing buffer and concentrated using a permanent magnet for SERS measurements.

### Nucleic acid detection in blood lysate

QuantiGene sample processing kit for blood samples (Thermofisher) was used for lysing blood samples. Cultured malaria-infected red blood cells (RBCs, ~400,000 parasites/μL) were used for testing the method. Non-infected RBCs or whole blood with and without synthetic target sequence spike-in were used as positive and negative controls (10 nM final target concentration for the positive control). Whole blood/RBCs working lysis mixture was prepared accordingly to the manufacturer protocol. Briefly, lysis mixture was pre-warmed at 37 C for 30 min. For 12 μL of whole blood or RBCs, 84 μL working lysis mixture containing of 32 μL lysis mixture, 50 μL RNase free water, and 2 μL of proteinase K was prepared and added, resulting in 96 μL solution. The solution was vortexed immediately for 30–60 seconds followed by incubation at 60 °C for 1 hour (preferentially under shaking).

After 1 hour, 7.68 μL of capture probe-functionalized amine magnetic beads and 11.52 μL reporter probe-functionalized nanorattles were added to each 96 μL lysate (blood lysate that was not used immediately was stored at −80 °C). The new mixture was incubated at 40 °C for 3–5 hours for sandwich hybridization.

Then, 30 μL aliquots was loaded into glass capillary tubes placed on the second-generation lab-on-a-stick device. The glass tubes had been previously loaded with four of 10 μL washing buffer solutions (SSC 5X, BSA 1%, SDS 0.02%, freshly prepared) separated by 3 μL mineral oil solutions. Magnetic beads were washed three times (50 cycles each) in the first three washing buffer solution before being moved to the final washing buffer solution for magnetic bead concentration and SERS signal measurement.

## Electronic supplementary material

Supporting Information

Video Capillary During Washing Step

Video Multiple Capillaries in Device

## References

[1] Niemz A, Ferguson TM, Boyle DS (2011). Point-of-care nucleic acid testing for infectious diseases. Trends in Biotechnology.

[2] Smith SJ, Nemr CR, Kelley SO (2017). Chemistry-Driven Approaches for Ultrasensitive Nucleic Acid Detection. J. Am. Chem. Soc..

[3] Zhu X (2015). Application of nanomaterials in the bioanalytical detection of disease-related genes. Biosens. Bioelectron..

[4] Miotke L, Barducci MC, Astakhova K (2015). Novel Signal-Enhancing Approaches for Optical Detection of Nucleic Acids-Going beyond Target Amplification. Chemosensors.

[5] Zheng Z, Luo YL, McMaster GK (2006). Sensitive and quantitative measurement of gene expression directly from a small amount of whole blood. Clinical Chemistry.

[6] D’Agata R (2011). Detection of genomic disorders in unamplified human genomic DNA using an ultrasensitive surface plasmon resonance imaging method. Int J Mol Med.

[7] Bertucci A (2015). Detection of unamplified genomic DNA by a PNA-based microstructured optical fiber (MOF) Bragg-grating optofluidic system. Biosens. Bioelectron..

[8] Cai H (2015). Optofluidic analysis system for amplification-free, direct detection of Ebola infection. Sci Rep.

[9] Mariani S, Scarano S, Spadavecchia J, Minunni M (2015). A reusable optical biosensor for the ultrasensitive and selective detection of unamplified human genomic DNA with gold nanostars. Biosens. Bioelectron..

[10] Mayr R (2016). A microfluidic platform for transcription- and amplification-free detection of zepto-mole amounts of nucleic acid molecules. Biosens Bioelectron.

[11] Xu Y, Zheng Z (2016). Direct RNA detection without nucleic acid purification and PCR: Combining sandwich hybridization with signal amplification based on branched hybridization chain reaction. Biosens. Bioelectron..

[12] Schlücker S (2014). Surface-Enhanced Raman Spectroscopy: Concepts and Chemical Applications. Angew. Chem. Int. Edit..

[13] Lane LA, Qian XM, Nie SM (2015). SERS Nanoparticles in Medicine: From Label-Free Detection to Spectroscopic Tagging. Chem. Rev..

[14] Kneipp K (1997). Single molecule detection using surface-enhanced Raman scattering (SERS). Phys. Rev. Lett..

[15] Nie SM, Emery SR (1997). Probing single molecules and single nanoparticles by surface-enhanced Raman scattering. Science.

[16] Vo-Dinh T (1998). Surface-enhanced Raman spectroscopy using metallic nanostructures. Trac-Trend. Anal. Chem..

[17] Vo-Dinh T (2013). Plasmonic nanoprobes: from chemical sensing to medical diagnostics and therapy. Nanoscale.

[18] Dinish, U. S., Balasundaram, G., Chang, Y. T. & Olivo, M. Actively Targeted *In Vivo* Multiplex Detection of Intrinsic Cancer Biomarkers Using Biocompatible SERS Nanotags. *Sci*. *Rep*. **4** (2014). 10.1038/srep04075 PMC3921631 24518045

[19] Isola NR, Stokes DL, Vo-Dinh T (1998). Surface enhanced Raman gene probe for HIV detection. Anal. Chem..

[20] Cao YWC, Jin RC, Mirkin CA (2002). Nanoparticles with Raman spectroscopic fingerprints for DNA and RNA detection. Science.

[21] Fabris L (2007). A heterogeneous PNA-based SERS method for DNA detection. J. Am. Chem. Soc..

[22] Kang T, Yoo SM, Yoon I, Lee SY, Kim B (2010). Patterned Multiplex Pathogen DNA Detection by Au Particle-on-Wire SERS Sensor. Nano Lett..

[23] Hu J (2010). Sub-attomolar HIV-1 DNA detection using surface-enhanced Raman spectroscopy. Analyst.

[24] He Y (2011). Silicon nanowires-based highly-efficient SERS-active platform for ultrasensitive DNA detection. Nano Today.

[25] Zhang ZL (2011). Mixed DNA-functionalized nanoparticle probes for surface-enhanced Raman scattering-based multiplex DNA detection. Chem Commun.

[26] Harper MM, Dougan JA, Shand NC, Graham D, Faulds K (2012). Detection of SERS active labelled DNA based on surface affinity to silver nanoparticles. Analyst.

[27] Zhang H, Harpster MH, Wilson WC, Johnson PA (2012). Surface-Enhanced Raman Scattering Detection of DNAs Derived from Virus Genomes Using Au-Coated Paramagnetic Nanoparticles. Langmuir.

[28] van Lierop D, Larmour IA, Faulds K, Graham D (2013). SERS Primers and Their Mode of Action for Pathogen DNA Detection. Anal. Chem..

[29] Wei X (2013). A Molecular Beacon-Based Signal-Off Surface-Enhanced Raman Scattering Strategy for Highly Sensitive, Reproducible, and Multiplexed DNA Detection. Small.

[30] Li J-M (2013). Highly Sensitive Detection of Target ssDNA Based on SERS Liquid Chip Using Suspended Magnetic Nanospheres as Capturing Substrates. Langmuir.

[31] Li M (2013). Plasmonic Nanorice Antenna on Triangle Nanoarray for Surface-Enhanced Raman Scattering Detection of Hepatitis B Virus DNA. Anal. Chem..

[32] Qi J (2014). Label-free, *in situ* SERS monitoring of individual DNA hybridization in microfluidics. Nanoscale.

[33] Hibbitts S (2014). Human papilloma virus genotyping by surface-enhanced Raman scattering. Analytical Methods.

[34] Su J (2017). Multicolor Gold Silver Nano-Mushrooms as Ready-to-Use SERS Probes for Ultrasensitive and Multiplex DNA/miRNA Detection. Anal. Chem..

[35] Pang Y (2016). Fe3O4@Ag magnetic nanoparticles for microRNA capture and duplex-specific nuclease signal amplification based SERS detection in cancer cells. Biosens. Bioelectron..

[36] Li YY (2016). Ultrasensitive Signal-On Detection of Nucleic Acids with Surface-Enhanced Raman Scattering and Exonuclease III-Assisted Probe Amplification. Anal. Chem..

[37] Fu X (2016). A SERS-based lateral flow assay biosensor for highly sensitive detection of HIV-1DNA. Biosens. Bioelectron..

[38] Ngo HT, Wang HN, Fales AM, Vo-Dinh T (2013). Label-Free DNA Biosensor Based on SERS Molecular Sentinel on Nanowave Chip. Anal. Chem..

[39] Ngo H, Wang H-N, Burke T, Ginsburg G, Vo-Dinh T (2014). Multiplex detection of disease biomarkers using SERS molecular sentinel-on-chip. Anal. Bioanal. Chem..

[40] Ngo HT (2014). DNA bioassay-on-chip using SERS detection for dengue diagnosis. Analyst.

[41] Laing S, Gracie K, Faulds K (2016). Multiplex *in vitro* detection using SERS. Chemical Society reviews.

[42] Chao J (2016). Nanostructure-based surface-enhanced Raman scattering biosensors for nucleic acids and proteins. J Mater Chem B.

[43] Ngo HT, Wang HN, Fales AM, Vo-Dinh T (2016). Plasmonic SERS biosensing nanochips for DNA detection. Anal Bioanal Chem.

[44] Zhang H, Harpster MH, Park HJ, Johnson PA (2011). Surface-Enhanced Raman Scattering Detection of DNA Derived from the West Nile Virus Genome Using Magnetic Capture of Raman-Active Gold Nanoparticles. Anal. Chem..

[45] Xu, H. *et al*. In *Raman Spectroscopy for Nanomaterials**Characterization* (ed ChallaS S. R. Kumar) Ch. 19, 531-551 (Springer Berlin Heidelberg, 2012).

[46] Donnelly T, Smith WE, Faulds K, Graham D (2014). Silver and magnetic nanoparticles for sensitive DNA detection by SERS. Chem Commun (Camb).

[47] Zhang JN, Joshi P, Zhou Y, Ding R, Zhang P (2015). Quantitative SERS-based DNA detection assisted by magnetic microspheres. Chem Commun.

[48] Kahraman, M., Mullen Emma, R., Korkmaz, A. & Wachsmann-Hogiu, S. In *Nanophotonics* Vol. 6, 831 (2017).

[49] Granger JH, Schlotter NE, Crawford AC, Porter MD (2016). Prospects for point-of-care pathogen diagnostics using surface-enhanced Raman scattering (SERS). Chemical Society reviews.

[50] Ngo HT, Gandra N, Fales AM, Taylor SM, Vo-Dinh T (2016). Sensitive DNA detection and SNP discrimination using ultrabright SERS nanorattles and magnetic beads for malaria diagnostics. Biosens. Bioelectron..

[51] Nanayakkara IA, Cao WD, White IM (2017). Simplifying Nucleic Acid Amplification from Whole Blood with Direct Polymerase Chain Reaction on Chitosan Microparticles. Anal. Chem..

[52] Ma YY (2010). Au@Ag Core-Shell Nanocubes with Finely Tuned and Well-Controlled Sizes, Shell Thicknesses, and Optical Properties. Acs Nano.

[53] Liu KK, Tadepalli S, Tian LM, Singamaneni S (2015). Size-Dependent Surface Enhanced Raman Scattering Activity of Plasmonic Nanorattles. Chem. Mater..

[54] Xia XH, Wang Y, Ruditskiy A, Xia YN (2013). 25th Anniversary Article: Galvanic Replacement: A Simple and Versatile Route to Hollow Nanostructures with Tunable and Well-Controlled Properties. Adv. Mater..

[55] Gandra N, Singamaneni S (2013). Bilayered Raman-Intense Gold Nanostructures with Hidden Tags (BRIGHTs) for High-Resolution Bioimaging. Adv. Mater..

[56] Skrabalak SE (2008). Gold Nanocages: Synthesis, Properties, and Applications. Accounts Chem Res.

[57] Gandra N (2016). Tunable and amplified Raman gold nanoprobes for effective tracking (TARGET): *in vivo* sensing and imaging. Nanoscale.

[58] Gracie K (2014). Simultaneous detection and quantification of three bacterial meningitis pathogens by SERS. Chem Sci.

[59] Moon GD (2011). A New Theranostic System Based on Gold Nanocages and Phase-Change Materials with Unique Features for Photoacoustic Imaging and Controlled Release. J. Am. Chem. Soc..

[60] Creecy, A., Russ, P. K., Solinas, F., Wright, D. W. & Haselton, F. R. Tuberculosis Biomarker Extraction and Isothermal Amplification in an Integrated Diagnostic Device. *Plos One* **10** (2015). 10.1371/journal.pone.0130260 PMC4488445 26132307

[61] Scherr, T. F., Ryskoski, H. B., Doyle, A. B. & Haselton, F. R. A two-magnet strategy for improved mixing and capture from biofluids. *Biomicrofluidics* **10** (2016). 10.1063/1.4946014 PMC4833749 27158286

[62] Adams, N. M. *et al*. Design criteria for developing low-resource magnetic bead assays using surface tension valves. *Biomicrofluidics* **7** (2013). 10.1063/1.4788922 PMC3562276 24403996

[63] Yamaguchi A, Matsuda K, Uehara M, Honda T, Saito Y (2016). A novel automated device for rapid nucleic acid extraction utilizing a zigzag motion of magnetic silica beads. Anal Chim Acta.

[64] Uehara M (2016). A Rapid and Automated Device for Purifying Nucleic Acids. Analytical Sciences.

[65] Shikida M, Sugito T, Okochi M, Honda H (2014). Droplet-based biochemical assay by magnetic wire manipulation between multiple droplets. Microsyst Technol.

[66] Gottheil R (2014). Moving the solid phase: a platform technology for cartridge based sandwich immunoassays. Biomed Microdevices.

[67] Wang, S. Q. *et al*. Micro-a-fluidics ELISA for Rapid CD4 Cell Count at the Point-of-Care. *Sci*. *Rep*. **4** (2014). 10.1038/srep03796 PMC3898414 24448112

[68] Chiou CH, Shin DJ, Zhang Y, Wang TH (2013). Topography-assisted electromagnetic platform for blood-to-PCR in a droplet. Biosens. Bioelectron..

[69] Otto TD (2010). New insights into the blood-stage transcriptome of Plasmodium falciparum using RNA-Seq. Mol Microbiol.

[70] Trager W, Jensen JB (1976). Human Malaria Parasites in Continuous Culture. Science.

[71] Hill HD, Mirkin CA (2006). The bio-barcode assay for the detection of protein and nucleic acid targets using DTT-induced ligand exchange. Nat Protoc.

