# Supplementary material for: Direct Detection of Unamplified Pathogen RNA in Blood Lysate using an Integrated Lab-in-a-Stick Device and Ultrabright SERS Nanorattles
Source: Sci Rep. 2018 Mar 6;8:4075. doi: 10.1038/s41598-018-21615-3 (PMC5840326; doi:10.1038/s41598-018-21615-3)
Supplement: Supplementary file 1 — Supporting Information [file 41598_2018_21615_MOESM1_ESM.pdf]

Supporting Documents

Direct Detection of unamplified Pathogen RNA in Blood Lysate using an Integrated Lab-in-a-Stick Device and Ultrabright SERS Nanorattles

Hoan T. Ngo[1,2], Elizabeth Freedman[1,3], Ren Abelard Odion[1,2], Pietro Strobbia[1,2], A. Swarnapali De Silva Indrasekara[1,2], Priya Vohra[1,2,4], Steve M. Taylor[1,3], and Tuan Vo-Dinh[1,2,5 (*)]

[1] Fitzpatrick Institute for Photonics, Duke University, Durham, NC 27708, USA
[2] Department of Biomedical Engineering, Duke University, Durham, NC 27708, USA
[3] Department of Medicine & Duke Global Health Institute, Duke University, Durham, NC 27708, USA
[4] Division of Head and Neck Surgery and Communication Sciences, Duke University, Durham, NC 27708, USA
[5] Department of Chemistry, Duke University, Durham, NC 27708, USA

Nanoparticles' extinction coefficient was measured using FLUOstar Omega microplate reader.

Nanoparticles' size distribution was acquired by analyzing TEM images using ImageJ.

SERS measurement by the handheld Raman reader:
Handheld Raman reader model C13560 provided by Hamamatsu Photonics K.K. was used. Laser wavelength was 785 nm, output power ~10 mW, and scan time 1 second.

SERS measurement by a lab-built system:
The lab-built SERS measurement system was composed of a 785 nm laser source (Rigaku Xantus-1), a fiber optic probe (InPhotonics RamanProbe), a spectrometer (Princeton Instruments Acton LS 785), and a CCD camera (Princeton Instruments PIXIS: 100BR_eXcelon). Laser power of the Xantus-1 was set at 200 mW and the CCD camera exposure time was set at 100 miliseconds, 10 accumulations (except Fig. 9 data were recorded using 1 second CCD camera exposure time, 10 accumulations).

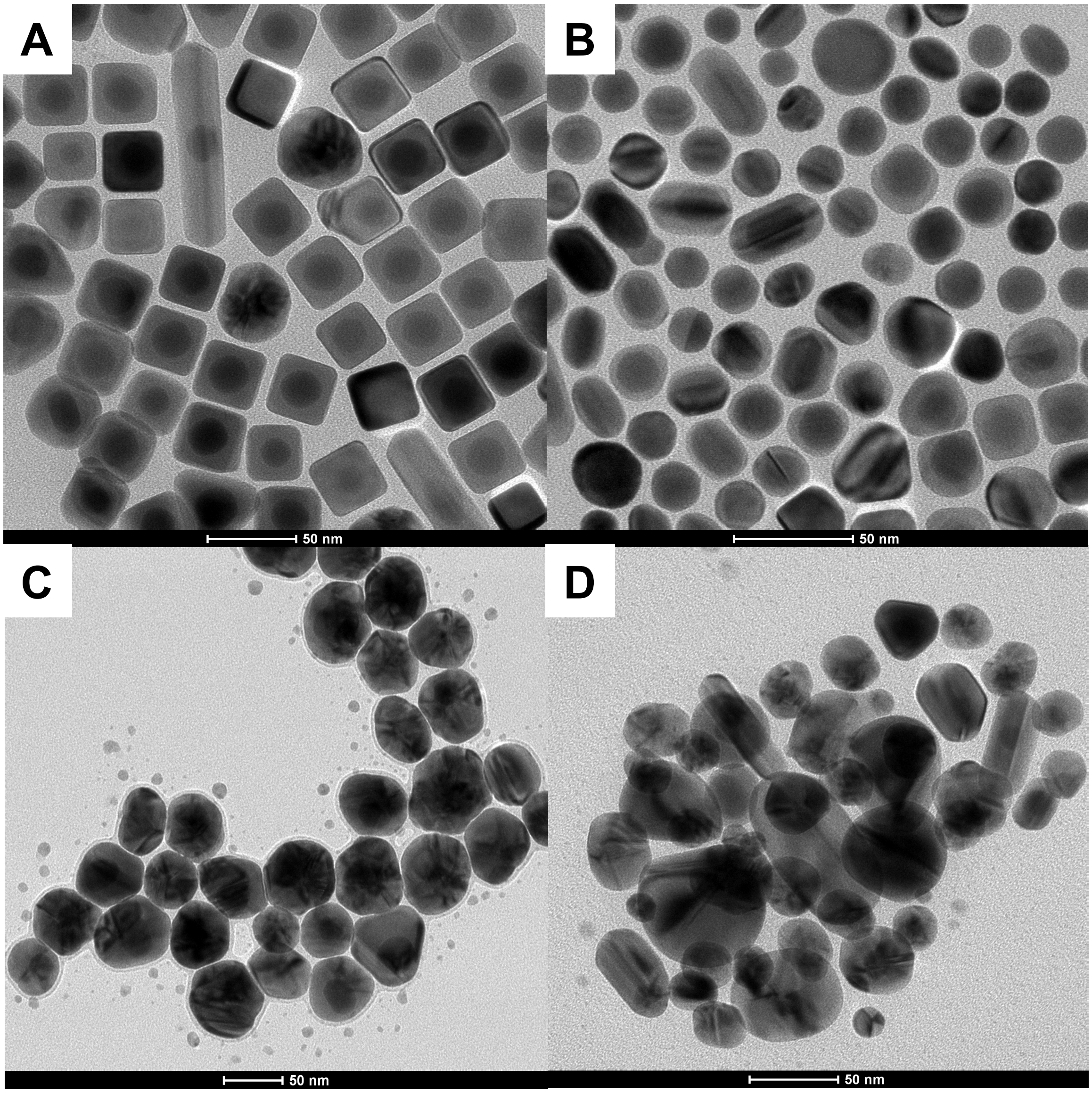

Figure S1: TEM images of AuNP@Ag prepared using different AuNP cores. (A) AuNP cores synthesized using growth solution containing CTAC and high ascorbic acid concentration (final concentration 15 mM) ; (B) AuNP cores synthesized using growth solution containing CTAC and low ascorbic acid concentration (final concentration 0.6 mM). (C) AuNP cores synthesized using citrate method followed by Ag shell coating without using CTAC [1]; (D) AuNP cores synthesized using citrate method followed by Ag shell coating using CTAC (similar to [2]).

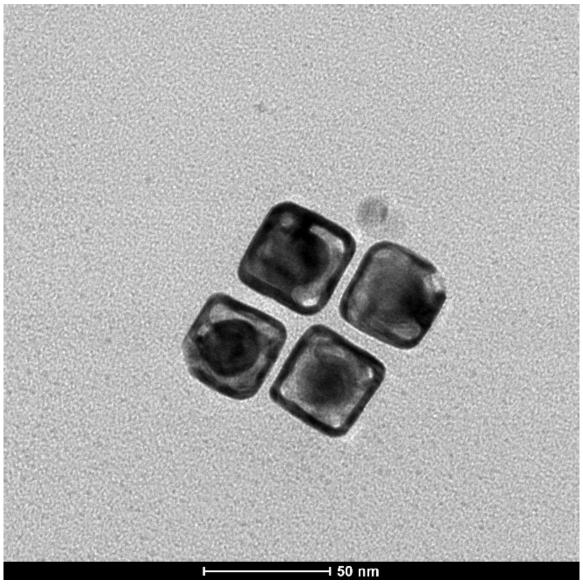

Figure S2: TEM image of AuNP@CubeCage (before Raman reporter loading).

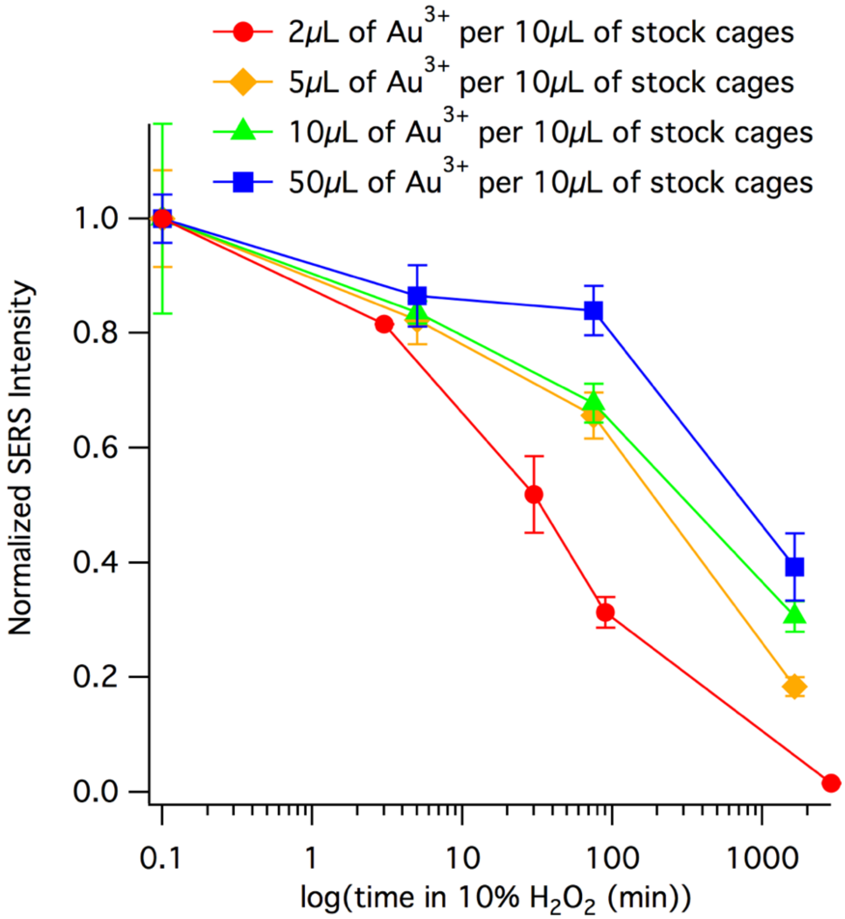

Figure S3: Normalized SERS intensity of nanorattles synthesized with different volume of Au^{3+} during final coating step as a function of time in 10% hydrogen peroxide (H_2O_2). The legend is the volume of Au^{3+} (~5.08 mM) per 10 ul of stock cages. Final coating of nanorattles was done in 10 ml batches, each used 50 µl of stock cages (see Materials and Method section for details).

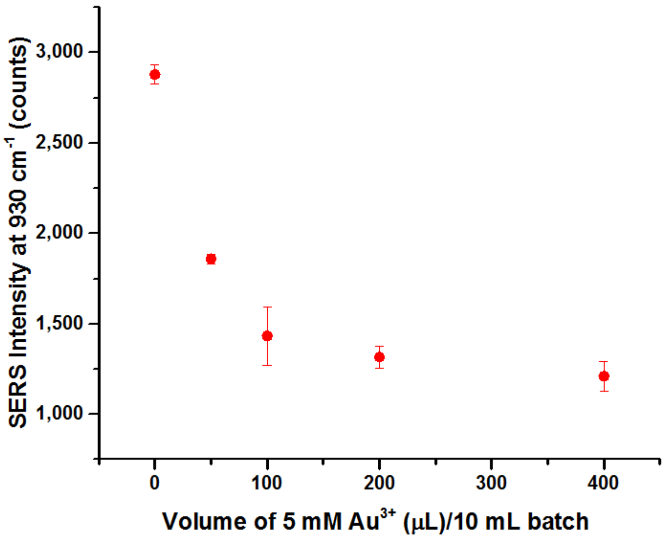

Fig. S4: SERS intensity of nanorattles with different amount of Au final coating.

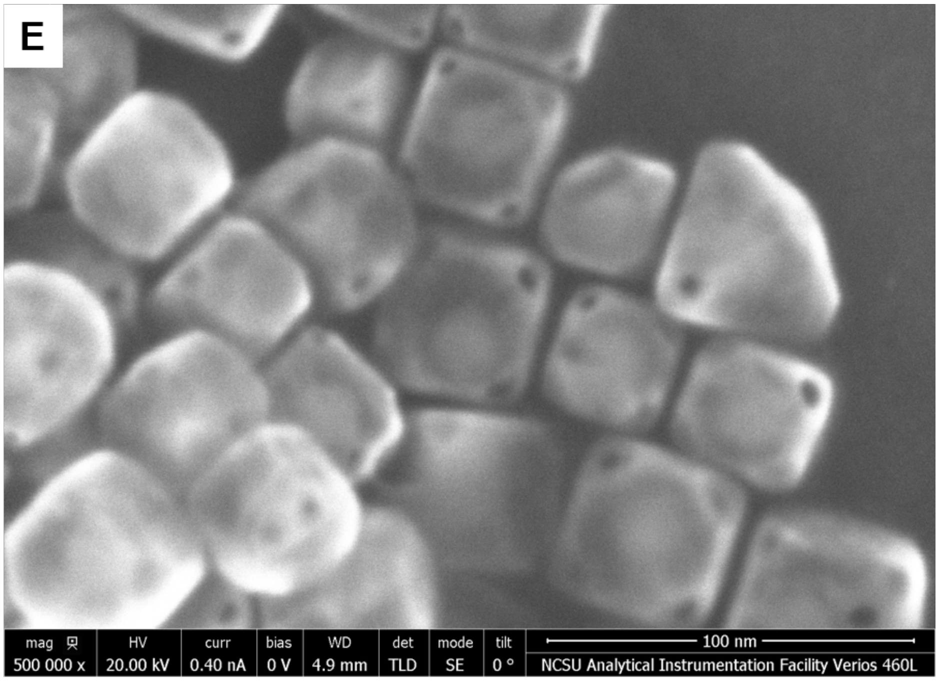

Figure S5: SEM images of AuNP@CubeCage. The cubic cages are clearly porous with holes at corners of the cubes.

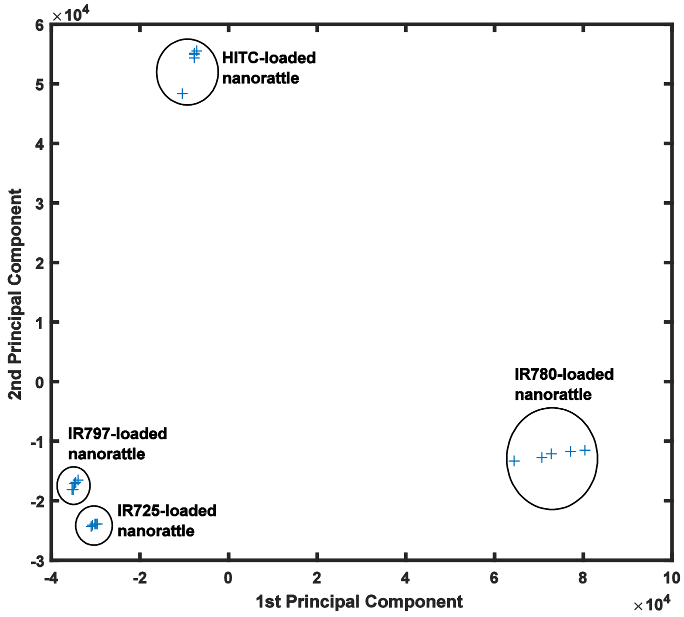

Figure S6. Principle component analysis of SERS spectra of SERS-encoded cube nanorattles loaded with different Raman reporters.

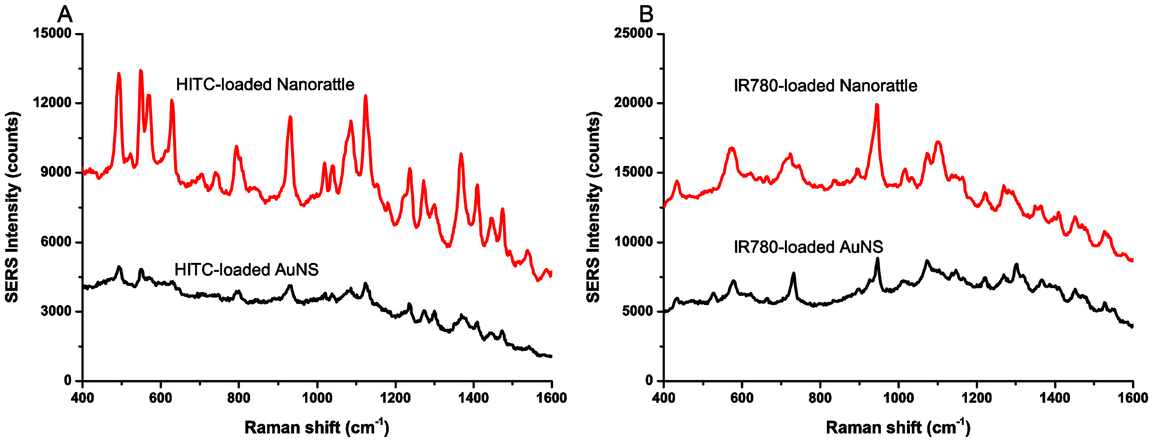

Figure S7: Comparison between nanorattles and gold nanostars loaded with (A) HITC Raman reporter, (B) IR780 Raman reporter.

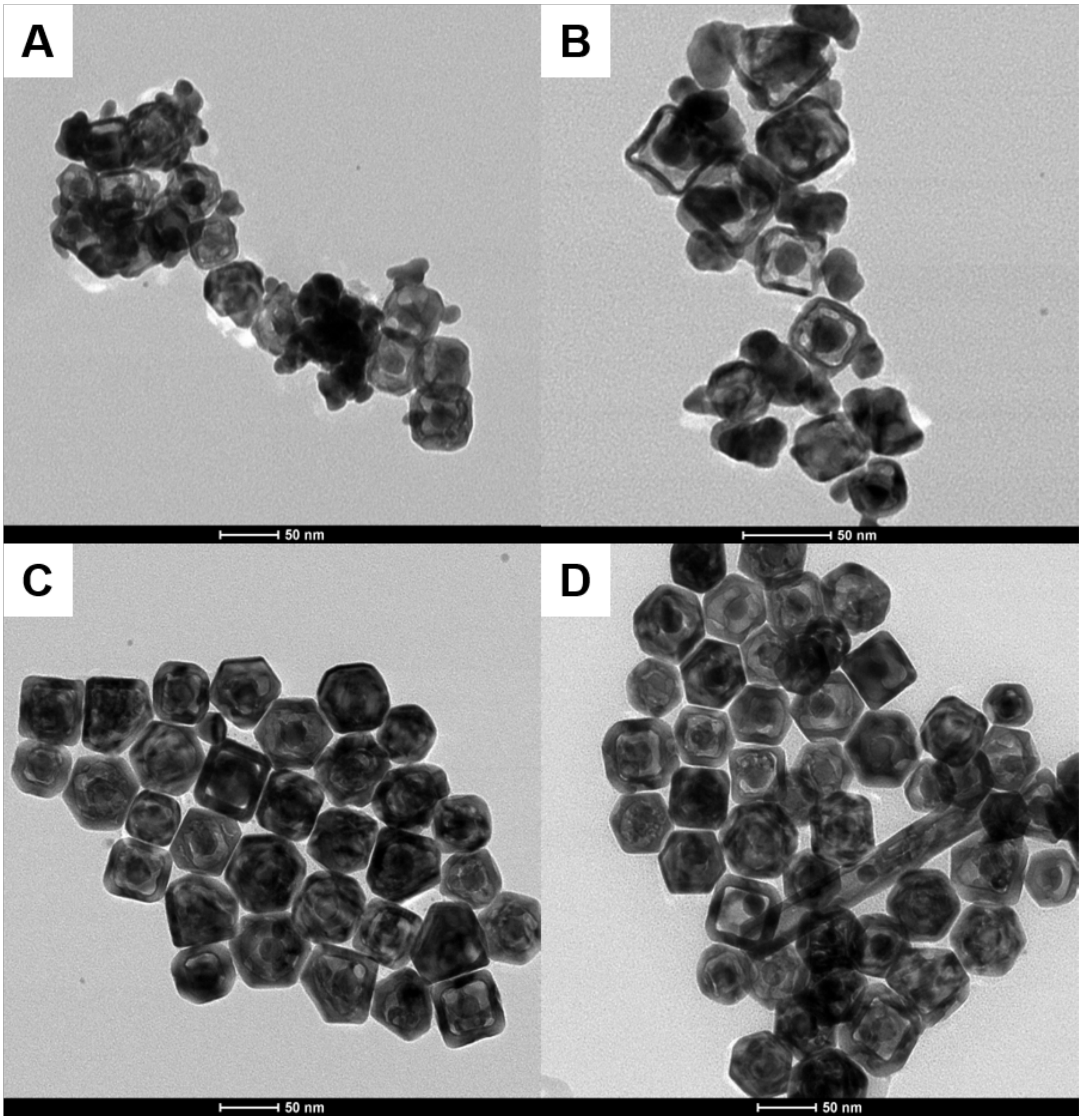

Figure S8: TEM images of nanorattles loaded with: (A) HITC, (B) DTTC), (C) IR780, (D) IR792 without using tetradecanol.

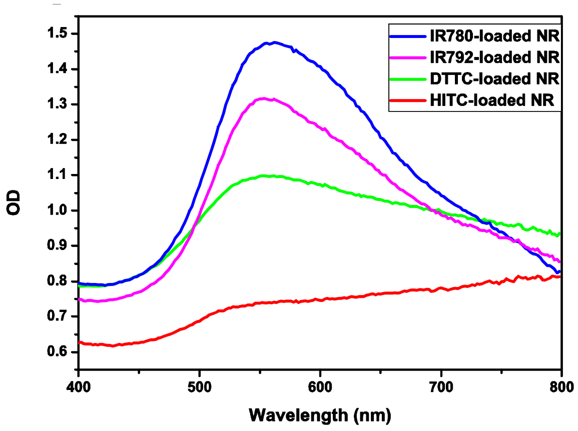

Figure S9: UV-Vis extinction spectra of cubic nanorattles loaded with different Raman reporters.

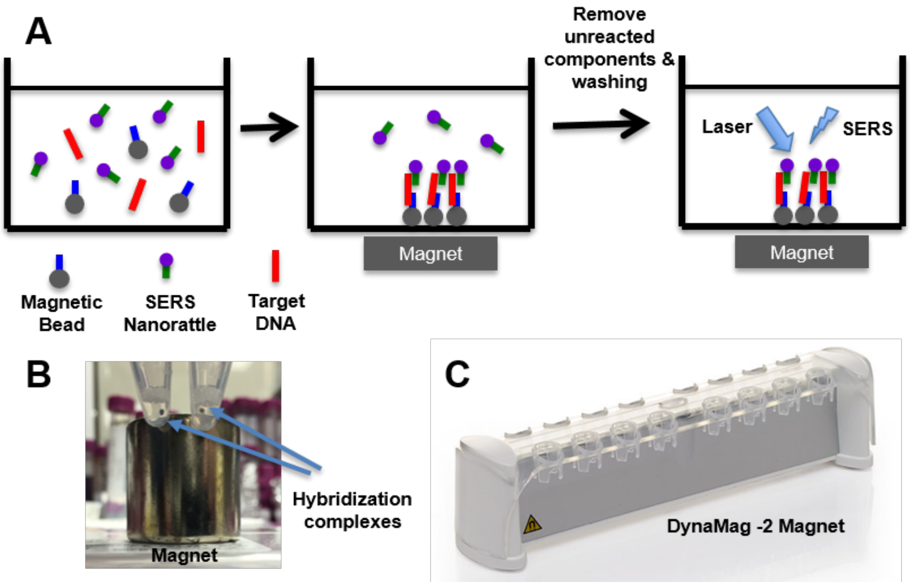

Figure S10: (A) Manual washing (B) Hybridization complexes in test tubes are concentrated by a permanent magnet. (C) DynaMag-2 can hold up to 16 test tubes simultaneously.

Table S1: Values from quantification curve of synthetic malaria DNA from Figure 7C.

Concentration (M)	SERS Intensity @930 rel. cm^{-1} (counts) [raw values]	Standard Deviation (counts)
10^{-14}	0.54×10^{3} [541]	0.03×10^{3}
10^{-13}	0.32×10^{3} [323]	0.05×10^{3}
10^{-12}	1.1×10^{3} [1096]	0.1×10^{3}
10^{-11}	10×10^{3} [10166]	2×10^{3}
10^{-10}	53×10^{3} [53058]	3×10^{3}
10^{-9}	56.8×10^{3} [56818]	0.3×10^{3}
10^{-8}	66×10^{3} [65924]	3×10^{3}

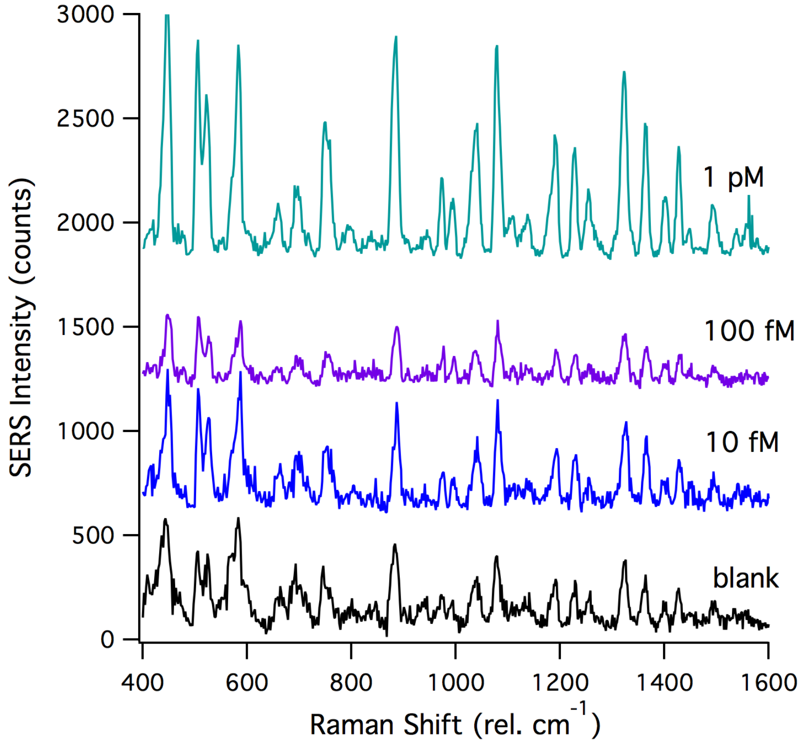

Figure S11: SERS spectra of blank, 10 fM, 100 fM and 1 pM of malaria synthetic DNA (extract of data shown in Figure 7B).

References

1. Jana, N.R., *Silver coated gold nanoparticles as new surface enhanced Raman substrate at low analyte concentration.* Analyst, 2003. **128**(7): p. 954-956.
2. Yang, J.H., et al., *Synthesis of Pt/Ag bimetallic nanorattle with Au core.* Scripta Materialia, 2006. **54**(2): p. 159-162.
